# Degradation‐Based Protein Profiling: A Case Study of Celastrol

**DOI:** 10.1002/advs.202308186

**Published:** 2024-04-25

**Authors:** Zhihao Ni, Yi Shi, Qianlong Liu, Liguo Wang, Xiuyun Sun, Yu Rao

**Affiliations:** ^1^ MOE Key Laboratory of Protein Sciences School of Pharmaceutical Sciences MOE Key Laboratory of Bioorganic Phosphorus Chemistry & Chemical Biology Tsinghua University Beijing 100084 China; ^2^ Changping Laboratory Beijing 102206 China

**Keywords:** celastrol, DBPP, PROTAC, toolbox

## Abstract

Natural products, while valuable for drug discovery, encounter limitations like uncertainty in targets and toxicity. As an important active ingredient in traditional Chinese medicine, celastrol exhibits a wide range of biological activities, yet its mechanism remains unclear. In this study, they introduced an innovative “Degradation‐based protein profiling (DBPP)” strategy, which combined PROteolysis TArgeting Chimeras (PROTAC) technology with quantitative proteomics and Immunoprecipitation‐Mass Spectrometry (IP‐MS) techniques, to identify multiple targets of natural products using a toolbox of degraders. Taking celastrol as an example, they successfully identified its known targets, including inhibitor of nuclear factor kappa B kinase subunit beta (IKKβ), phosphatidylinositol‐4,5‐bisphosphate 3‐kinase catalytic subunit alpha (PI3Kα), and cellular inhibitor of PP2A (CIP2A), as well as potential new targets such as checkpoint kinase 1 (CHK1), O‐GlcNAcase (OGA), and DNA excision repair protein ERCC‐6‐like (ERCC6L). Furthermore, the first glycosidase degrader is developed in this work. Finally, by employing a mixed PROTAC toolbox in quantitative proteomics, they also achieved multi‐target identification of celastrol, significantly reducing costs while improving efficiency. Taken together, they believe that the DBPP strategy can complement existing target identification strategies, thereby facilitating the rapid advancement of the pharmaceutical field.

## Graphical abstract

In this study, an innovative “Degradation‐based protein profiling (DBPP)” strategy is introduced, which combined PROteolysis TArgeting Chimera (PROTAC) technology with quantitative proteomics and Immunoprecipitation‐Mass Spectrometry (IP‐MS) techniques, to identify multiple targets of natural products using a toolbox of degraders. Taking Celastrol as an example, they have successfully identified its three known targets and three potential new targets. Furthermore, this strategy is optimized by employing a mixed PROTAC toolbox in quantitative proteomics.

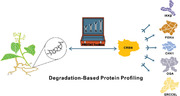

## 1 Introduction

### 1.1 Natural Products and Target Identification

Natural products possess unique chemical structures and diverse biological activities, rendering them a treasury of hit compounds for drug development. Statistics indicated that approximately two‐thirds of small‐molecule drugs had been derived to varying degrees from natural products between 1981 and 2019.^[^ ^1^ ^]^ Phenotypic screening techniques allow scientists to gain deeper insights into cellular phenotypes, signaling pathways, and disease phenotypes influenced by natural products. However, the precise identification of targets for natural products remains one of the most significant challenges in the field.^[^ ^2^ ^]^

**Figure 1:**
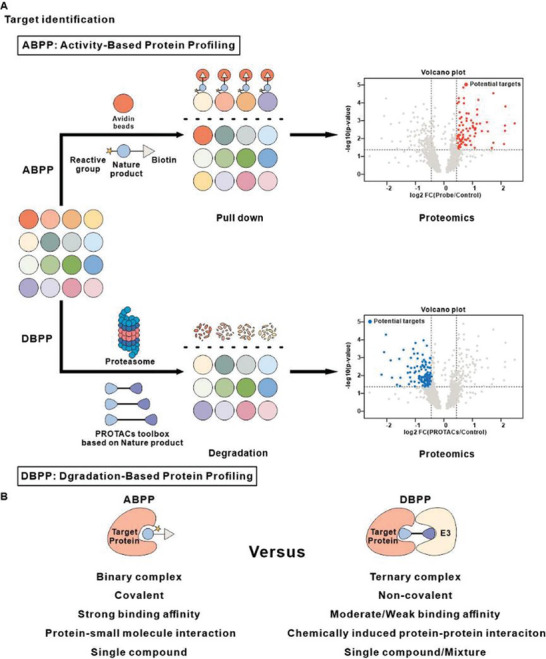
The comparison between ABPP and DBPP. A) A schematic diagram of comparison between DBPP strategy and ABPP strategy. The ABPP strategy uses covalent probes to pull down target proteins, and proteins upregulated compared to the control group in proteomics data are regarded as potential targets. The DBPP strategy degrades the target proteins through the non‐covalent PROTAC toolbox, and proteins downregulated compared to the control group in proteomics data are regarded as potential targets. B) The feature comparison between the two strategies.

Currently, the most commonly used approach for target identification is chemical proteomics, with Activity‐Based Protein Profiling (ABPP) emerging as a powerful tool in recent years to study the interactions between small molecules and proteins. The core principle of this strategy lies in designing activity‐based probes that covalently react with amino acid residues in the active centers of proteins within the proteome.^[^ ^3^ ^]^ The working principle of ABPP involves initially using probes to covalently label target proteins, followed by attaching fluorescent or biotin moieties to proteins. Ultimately, potential targets are identified through gel fluorescence imaging or quantitative proteomics (**Figure** **1**A). ABPP has been widely applied in the target identification of natural products and small molecule drugs, such as bile acids^[^ ^4^ ^]^ and aspirin.^[^ ^5^ ^]^ However, this strategy has certain limitations: 1) the introduction of crosslinking and reporter moieties may affect the binding affinity of natural products; 2) the strategy heavily relies on the binding affinity between small molecules and proteins, requiring the external introduction of photo‐crosslinking moieties for non‐covalent small molecules, but these technical requirements are often overly stringent and lack universality; 3) non‐specific binding of photo‐reactive moieties can affect the accuracy of results;^[^ ^6^ ^]^ 4) Only a small proportion of the human proteins have a well‐defined structure and many of these proteins do not have a catalytic function, let alone a good ligand or a discernible ligand‐binding site. Additionally, there are some label‐free strategies as supplementary methods, including protein microarrays, thermal proteome profiling (TPP), and limited proteolysis coupled with mass spectrometry (LiP‐MS). However, these techniques have their respective limitations, especially in identifying weak binding targets. Moreover, natural products typically target multiple proteins, and their binding affinities are usually moderate or weak. Consequently, there is currently a deficiency in an efficient target identification strategy based on non‐covalent binding.

PROTAC (Proteolysis‐targeting Chimera) is a cutting‐edge chemical biology technology that has emerged in recent years. It operates by hijacking the ubiquitin‐proteasome system using a bifunctional small molecule that can bind non‐covalently to both a target protein and an E3 ubiquitin ligase, inducing ubiquitination and subsequent degradation of the target protein. So far, this technology has successfully facilitated the degradation of more than 130 different proteins.^[^ ^7^ ^]^ In this work, we have developed a novel multi‐target identification strategy called “Degradation‐based protein profiling (DBPP)” by combining PROTAC technology with quantitative proteomics and IP‐MS techniques. Within this strategy, proteins downregulated compared to the control group in proteomics data are regarded as potential targets of natural products. Unlike the ABPP strategy, the DBPP strategy relies on chemically induced protein‐protein interaction (PPI) rather than protein‐small molecule interaction. Therefore, theoretically, moderate or weak binding proteins can be efficiently degraded through the DBPP strategy. At the same time, based on the formation of ternary complex between binding protein, PROTAC molecule and E3 ligase, we can further utilize the IP‐MS experiment to pull down and identify potential target proteins. To conceptually validate this strategy, we used celastrol as an illustrative example (Figure 1).

### 1.2 Celastrol

Celastrol is a quinone‐containing triterpenoid compound isolated from Tripterygium wilfordii, recognized as one of the top five promising traditional Chinese medicine molecules to be developed into modern drugs. It exhibits a wide range of biological activities in tumor therapy,^[^ ^8^ ^]^ immune inflammation,^[^ ^9^ ^]^ neurodegenerative diseases,^[^ ^10^ ^]^ and metabolic disorders.^[^ ^11^ ^]^ Although some studies have revealed molecular targets and signaling pathways of celastrol, such as the PI3K/AKT pathway,^[^ ^12^ ^]^ NF‐κB pathway,^[^ ^13^ ^]^ STAT3,^[^ ^14^ ^]^ HSP90,^[^ ^15^ ^]^ and proteasome,^[^ ^16^ ^]^ among others,^[^ ^17^ ^]^ its exact mechanism of action remains incompletely understood. Moreover, challenges like poor water solubility, low bioavailability, narrow therapeutic window, and potential adverse reactions severely hinder its clinical application.^[^ ^18^ ^]^ Therefore, the main challenge in celastrol development lies in efficiently identifying its targets and subsequently conducting rational structural modifications to enhance its activity or reduce its toxicity.

**Figure 2:**
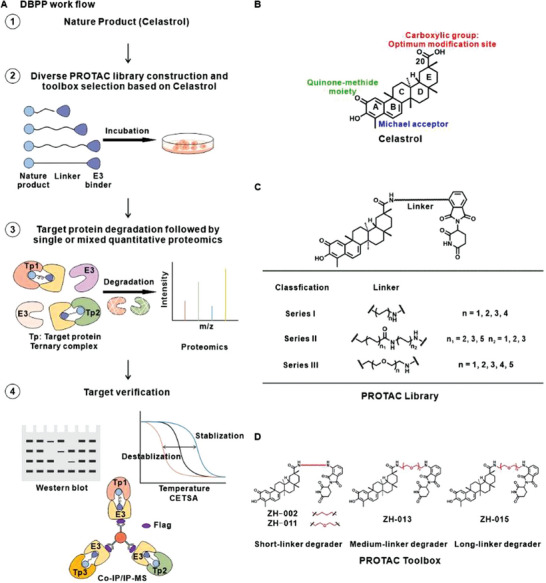
DBPP work flow and PROTAC library construction based on celastrol. A) DBPP work flow. Compound library construction based on natural products and selection of representative toolbox molecules, followed by quantitative proteomics and final verification of potential targets. B) The structure and modification sites of celastrol. C) The PROTAC library based on celastrol. D) The structure of PROTAC toolbox ZH‐002, ZH‐011, ZH‐013, ZH‐015 with varying types and lengths of linkers.

In this study, we employed the DBPP strategy to identify celastrol's targets. First, we designed and synthesized a diverse library of PROTAC based on celastrol. Then we selected a set of toolbox molecules considering factors such as linker type, length, and cellular phenotype. Subsequently, through the application of quantitative proteomics and other biological techniques, we successfully identified and validated multiple targets of celastrol, including its known targets inhibitor of nuclear factor kappa B kinase subunit beta (IKKβ) and hosphatidylinositol‐4,5‐bisphosphate 3‐kinase catalytic subunit alpha (PI3Kα), as well as potential new targets checkpoint kinase 1 (CHK1) and O‐GlcNAcase (OGA) (**Figure** **2**A). We speculated that CHK1 was a potential target for celastrol‐induced toxicity, while OGA might be involved in the functions of celastrol in metabolism and neurodegenerative diseases.^[^ ^19^ ^]^ Furthermore, by comparing the quantitative proteomics data and IP‐MS data of ZH‐011, we not only validated the previously identified targets but also uncovered another known target, cellular inhibitor of PP2A (CIP2A) and a potential new target, DNA excision repair protein ERCC‐6‐like (ERCC6L). We believed that the integration of those two proteomic techniques can significantly enhance the accuracy of target identification. Finally, we found that utilizing a mixed toolbox in quantitative proteomics further improved the efficiency of this strategy.

## 2 Results

### 2.1 Construction and Phenotypic Screening of the PROTAC Library Based on Celastrol

Previous studies on the structure‐activity relationship (SAR) of celastrol have mainly focused on the quinone methyl group in ring A, the Michael acceptor in ring B, and the carboxyl group in ring E^[^ ^20^ ^]^ (Figure 2B). We selected the C‐20 carboxyl group that does not affect the compound's activity as an attachment site for the linker and chose pomalidomide as the binder for the E3 ligase Cereblon (CRBN). Based on our understanding of PROTAC technology, the orientations, types, and lengths of the linkers significantly influence the selectivity and activity of PROTAC molecules. Therefore, we constructed a diverse library of PROTAC molecules derived from celastrol, incorporating linkers comprised of alkyl chains or polyethylene glycol (PEG) chains with varying lengths (Figure 2C; Table S1, Supporting Information).

Firstly, we conducted a phenotypic screening for cell proliferation inhibition on Jurkat cells (Figure S1, Supporting Information). Among the short‐chain PROTAC, ZH‐002 with an alkyl chain (IC_50_ = 0.25 µm) and ZH‐011 with a PEG chain (IC_50_ = 0.40 µm) exhibited relatively stronger proliferation inhibition. Considering the diverse biological activities of celastrol and the possibility that PROTAC molecules with varying linker lengths may degrade different targets, we further selected the medium‐chain degrader ZH‐013 (IC_50_ = 0.69 µm) and the long‐chain degrader ZH‐015 (IC_50_ = 0.56 µm), based on ZH‐002 and ZH‐011, to compose a PROTAC toolbox for celastrol target identification (Figure 2D).

### 2.2 Tandem Mass Tag (TMT)‐Labeled Quantitative Proteomics

Next, we conducted TMT‐labeled quantitative proteomics experiments on Jurkat cells treated with the PROTAC toolbox (ZH‐002, ZH‐011, ZH‐013, and ZH‐015), respectively. Each degrader was subjected to three biological replicates. To balance the degradation activity and selectivity of PROTAC molecules, we used a moderate concentration of 500 nm. In order to investigate direct rather than indirect protein degradation, cells were treated for only 8 h.

**Figure 3:**
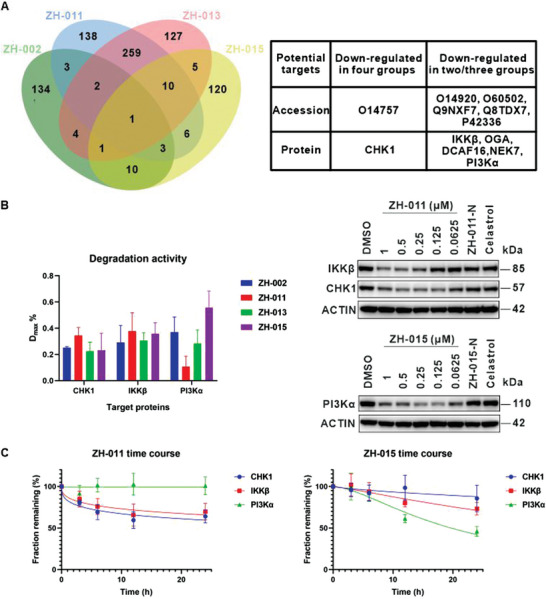
The PROTAC toolbox downregulated PI3Kα, IKKβ, and CHK1. A) The Venn diagram showed the individually downregulated proteins by each toolbox molecule and the intersection of downregulated proteins among four toolbox molecules. The significance analysis was conducted by two‐tailed unpaired Student's *t*‐tests using Microsoft Excel spreadsheets with basic statistical program (biological replicates; n = 3, *p* value <0.05). The table showed the potential targets we focused on. B) On the left side, the maximum degradation efficiency of three proteins by each toolbox molecule was shown. Jurkat cells were treated with the indicated concentrations of toolbox molecule for 8 h before WB and grayscale quantification. Data are represented as mean ± SD, n = 3. On the right side, dose‐dependent degradation experiments of the optimal molecules for each target were assessed by WB, the Jurkat cells were treated with ZH‐011, ZH‐015, ZH‐011‐N (500 nm), ZH‐015‐N (500 nm), celastrol (500 nm) for 8 h. C) The time‐dependent degradation experiments of all three proteins. Jurkat cells were treated separately with ZH‐011 and ZH‐015 at a concentration of 500 nm for the indicated time before WB and grayscale quantification. Data are represented as mean ± SD, n = 3.

We analyzed the proteomic data following these principles: 1) We used a PROTAC group/DMSO group ratio of 0.88 as the cutoff to remove any non‐specifically degraded target proteins (*p* value <0.05). 2) To improve the accuracy of target identification, we only further analyzed downregulated proteins identified in at least two out of the four groups. 3) To eliminate interference from proteins downregulated by the ligand, we excluded proteins that were also downregulated in the celastrol group. Combining the analysis results with the known biological functions of celastrol, we ultimately focused on six proteins (IKKβ, PI3Kα, CHK1, OGA, DCAF16, and NEK7) (**Figure** **3**A; Extended Data S1, Supporting Information).

In accordance with credibility, we classified the identified targets into two categories: those with the highest credibility, including PI3Kα, IKKβ, and CHK1, and those with high credibility, including OGA, NEK7, and DCAF16. Abundant literature has previously established the association between celastrol and the PI3K/Akt as well as the NF‐κB signaling pathways. PI3Kα and IKKβ, serving as key proteins in these two pathways, were considered to have the highest credibility. Additionally, CHK1, as the only protein downregulated in all four groups, also held the highest credibility.

### 2.3 Target Verification by Western Blot

To validate the results from proteomic analysis, we conducted western blot (WB) experiments. First, we synthesized negative control compounds for the toolbox, namely ZH‐002‐N, ZH‐011‐N, ZH‐013‐N, and ZH‐015‐N, which have an ethyl group at the NH position of glutarimide to prevent their binding to CRBN (Table S1, Supporting information). Our initial validation mainly focused on three proteins, IKKβ, PI3Kα, and CHK1. WB results showed that all three targets exhibited dose‐dependent and time‐dependent degradation (Figure 3B,C; Figures S2 and S3, Supporting Information).

Concerning IKKβ, all four compounds caused significant degradation. Regarding CHK1, compounds with shorter linkers exhibited better degradation activity, with ZH‐011 inducing the most efficient degradation (Dmax = 35%). As for PI3Kα, compounds with longer linkers showed better degradation activity, with ZH‐015 identified as the optimal degrader (Dmax = 56%). Notably, ZH‐015 showed a significant “hook effect” at 500 nm and did not affect the protein levels of PI3Kβ or PI3Kγ (Figure S4A, Supporting Information). As anticipated, ZH‐013, with a medium‐length linker, exhibited moderate degradation activity for each protein, and these structure‐activity relationships were also confirmed in the evaluation of the entire compound library (Figure S4B, Supporting Information). Even more intriguingly, ZH‐011 demonstrated the most effective CHK1 degradation activity with virtually no impact on the protein level of PI3Kα, while ZH‐015 displayed optimal PI3Kα degradation activity with only a slight downregulation of the CHK1 protein level (Figure 3C; Figure S2 and S3, Supporting Information). Consequently, we have selected these two molecules as tool compounds for further study. It is worth noting that neither the negative control nor celastrol caused downregulation of the target proteins.

Furthermore, we performed a brief evaluation of three other proteins with high confidence using WB. Due to the absence of commercially available DCAF16 antibodies, we constructed a stably expressed Flag‐DCAF16 293T cell line. The WB results indicated that all the proteins were downregulated by PROTAC molecules, whereas the negative control or celastrol did not induce downregulation (Figure S5A,B, Supporting Information). For OGA, short‐chain degraders could significantly induce its downregulation, with ZH‐011 demonstrating the optimal activity. The downregulation of DCAF16, an incompletely characterized Cullin‐RING E3 ubiquitin ligase, suggests that celastrol may be a new E3 ligase recruiter. The downregulation of NEK7, which is involved in the activation of NLRP3 inflammasome, may account for celastrol's anti‐inflammatory activity. However, the validation of these two targets, NEK7 and DCAF16, was temporarily halted in this study due to the absence of an optimal molecule for NEK7 and the unavailability of commercial antibodies for DCAF16. In summary, our subsequent work primarily focused on the target proteins PI3Kα, IKKβ, CHK1, and OGA.

### 2.4 IKKβ and PI3Kα were Identified as the Targets of Celastrol

Previous studies have demonstrated that celastrol can directly inhibit the kinase activities of PI3K^[^ ^12^ ^]^ and IKKβ.^[^ ^21^ ^]^ Moreover, multiple studies have associated the impact of celastrol on the PI3K/Akt and NF‐κB signaling pathways with its anti‐tumor and anti‐inflammatory activities.^[^ ^21^, ^22^ ^]^ Therefore, we attempted to establish a target validation system using these two known targets.

**Figure 4:**
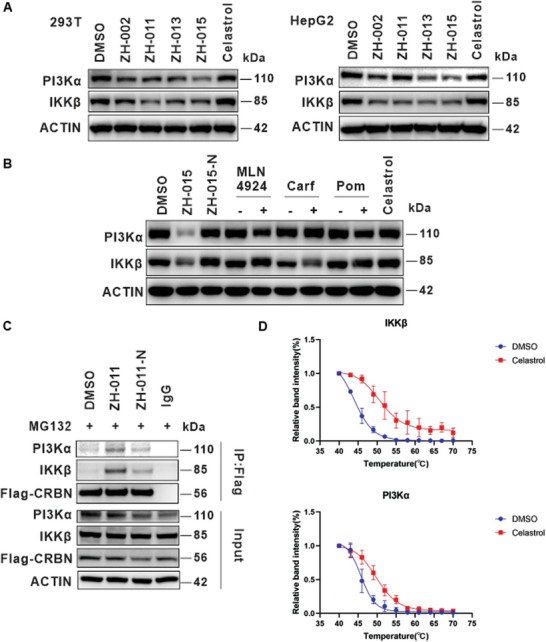
IKKβ and PI3Kα were identified as the targets of celastrol. A) Degradation activity of PROTAC toolbox on IKKβ and PI3Kα in 293T and HepG2 cells. Cells were treated with DMSO, ZH‐002 (500 nm), ZH‐011 (500 nm), ZH‐013 (500 nm), ZH‐015 (500 nm), and celastrol (500 nm) for 8 h before WB. B) The rescue experiment of IKKβ and PI3Kα. Carf and Poma are abbreviations for carfilzomib and pomalidomide. Jurkat cells were pretreated with MLN4924 (500 nm), carfizomib (500 nm), and pomalidomide (20 µm) for 2 h, followed by treatment of ZH‐015 (500 nM) for 8 h before WB. C) Co‐immunoprecipitation of endogenous IKKβ and PI3Kα from Flag‐CRBN in 293T cells after pretreatment with MG132 (5 µm) for 1 h, followed by treatment of DMSO, ZH‐011 (10 µm), ZH‐011‐N (10 µm) separately for 3 h. D) Melt and shift curve of IKKβ and PI3Kα in Jurkat cells treated with DMSO control (blue) and 50 µm celastrol (red). Data are represented as mean ± SD, n = 3.

First, we validated the universality of the PROTAC toolbox for the degradation of PI3Kα and IKKβ in 293T and HepG2 cells (**Figure** **4**A). The results showed that the PROTAC toolbox maintained its degradation activity in both cell lines, with ZH‐015 still inducing the most significant degradation of PI3Kα, and several molecules, including ZH‐015, causing obvious downregulation of IKKβ. Therefore, ZH‐015 was chosen as a tool compound to investigate the degradation mechanisms of IKKβ and PI3Kα. As shown in Figure 4B, the NEDD8‐activating enzyme inhibitor MLN4924 and the proteasome inhibitor carfilzomib significantly blocked the degradation of the target proteins. Additionally, the addition of CRBN binder pomalidomide effectively prevented the degradation of the target proteins, and the negative control also failed to induce degradation of the target proteins. In the CRBN‐knockout (CRBN^−/−^) Jurkat cells, ZH‐015 was unable to degrade PI3Kα and IKKβ (Figure S6B, Supporting Information). Overall, these results revealed that the degradation of these two proteins was mediated through the ubiquitin‐proteasome pathway.

Subsequently, we performed Co‐immunoprecipitation (Co‐IP) experiments on IKKβ and PI3Kα. As PROTAC molecules can induce the formation of ternary complexes, we aimed to demonstrate through Co‐IP whether our degraders have direct interactions with the target proteins. We initially conducted a preliminary experiment on Flag‐CRBN 293T cells using two molecules with optimal single‐target degradation activity, ZH‐011 and ZH‐015. We observed that ZH‐015 exhibited better pull‐down efficacy on PI3Kα compared to ZH‐011(Figure S7A, Supporting Information), which correlated positively with molecule's degradation activity. However, considering that ZH‐011 displayed better degradation activity on CHK1 and OGA, we decided to proceed with ZH‐011 for the subsequent Co‐IP experiments. Then we assessed the pull‐down efficiency of ZH‐011 on the target protein at different concentrations and found that higher concentrations of ZH‐011 yielded better results (Figure S7B, Supporting Information). Therefore, we set the concentration of ZH‐011 at 10 µm for the ensuing experiments. As shown in Figure 4C, we observed that both target proteins were effectively pulled down in the PROTAC group rather than in the control group. This result indicated that ZH‐011 could form stable ternary complexes with CRBN and the target proteins.

To further confirm that IKKβ and PI3Kα are direct targets of celastrol, we conducted the cellular thermal shift assay (CETSA). Each experiment was repeated three times, and thermal melting curves were generated (Figure S8, Supporting Information). As shown in Figure 4D, celastrol significantly enhanced the thermal stability of both proteins, indicating direct interactions between celastrol and IKKβ, as well as PI3Kα.

Inspired by these two target proteins, we turned our attention to another known target, NR4A1, as reported in the literature.^[^ ^23^ ^]^ Because the expression of NR4A1 in Jurkat cells requires stimulation and induction,^[^ ^24^ ^]^ quantitative proteomics failed to identify this protein. Therefore, we evaluated the degradation activity of our toolbox molecules against NR4A1 in HeLa cells. The results demonstrated that our PROTAC toolbox molecules could also effectively induce the degradation of NR4A1 (Figure S5C, Supporting Information).

### 2.5 CHK1 Might Be One of the Toxicity Targets of Celastrol

**Figure 5:**
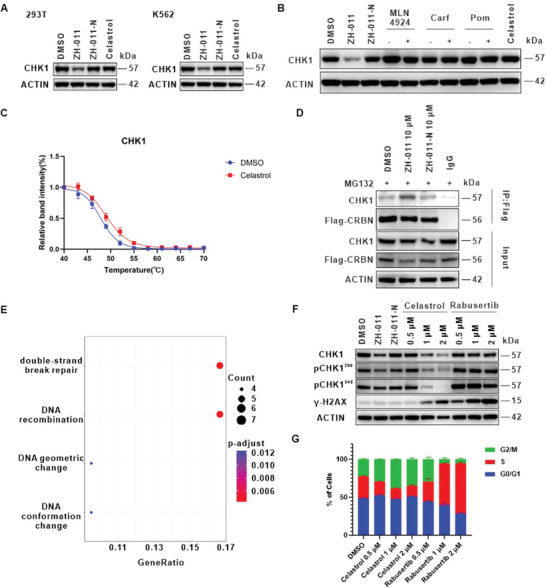
CHK1 might be one of the toxicity targets of celastrol. A) Degradation activity of ZH‐011 on CHK1 protein in 293T and K562 cells. Cells were treated with DMSO, ZH‐011 (500 nm), ZH‐011‐N (500 nm), and celastrol (500 nm) for 24 h before WB. B) The rescue experiment of CHK1. Carf and Poma are abbreviations for carfilzomib and pomalidomide. Jurkat cells were pretreated with MLN4924 (500 nm), carfilzomib (500 nm), and pomalidomide (20 µm) for 2 h, followed by treatment of ZH‐011 (500 nm) for 8 h before WB. C) Melt and shift curve of CHK1 in Jurkat cells treated with DMSO control (blue) and 50 µm celastrol (red). Data are represented as mean ± SD, n = 3. D) Co‐immunoprecipitation of endogenous CHK1 from Flag‐CRBN in 293T cells after pretreatment with MG132 (5 µm) for 1 h, followed by treatment of DMSO, ZH‐011 (10 µm), ZH‐011‐N (10 µm) separately for 3 h.E) GO analysis of the significantly affected proteins in the celastrol group. F) The protein levels of phospho‐Chk1 (Ser 296, Ser 345), and γH2AX assessed by WB after Jurkat cells were treated with DMSO, ZH‐011 (0.5 µm), ZH‐011‐N (0.5 µm), celastrol (2, 1, and 0.5 µm) and rabusertib (2, 1, and 0.5 µm) for 24 h, respectively. G) Jurkat cells were treated with DMSO, celastrol (2, 1, and 0.5 µm), and rabusertib (2, 1, and 0.5 µm) for 24 h. The cell cycle was assayed by FACS with propidium iodide (PI) staining. The cell cycle phase distribution was analyzed by FlowJo software. Data are represented as mean ± SD, n = 3.

CHK1 plays a crucial role in regulating cell cycle, DNA damage checkpoint control, embryonic development, and tumor suppression. This protein may account for the toxicity of celastrol, so we focused on the verification of CHK1. Unlike ZH‐015, ZH‐011 showed the weakest PI3Kα degradation activity and the strongest CHK1 degradation activity, making it suitable for subsequent experiments. We validated the wide applicability of ZH‐011 on CHK1 in both 293T and K562 cell lines and performed rescue experiments to verify the mechanism of CHK1 degradation (**Figures** **5**A,B; S6A, Supporting Information).

Subsequently, we conducted Co‐IP and CETSA experiments on CHK1 protein (Figure 5C,D). As expected, ZH‐011 successfully pulled down the CHK1, and celastrol enhanced the thermal stability of CHK1. Although the effect of celastrol on the thermal shift of CHK1 was not pronounced, it still indicated a direct interaction between celastrol and CHK1.^[^ ^25^ ^]^

To further elucidate the association between CHK1 and celastrol, we performed a Gene Ontology (GO) analysis on the proteins affected by celastrol (Figure 5E). When analyzing the proteomic data of celastrol, we used a celastrol group/DMSO group ratio of 0.88 or 1.14 (*p* value <0.05) as the cutoff to exclude non‐significantly affected targets, and only proteins identified in at least two out of three replicates were used for GO analysis. The result revealed that DNA double‐strand break repair was the most significantly enriched biological process, which is closely related to the functions of both celastrol^[^ ^26^ ^]^ and CHK1.

Next, we investigated the CHK1‐related downstream signaling pathways affected by celastrol. We found that under high‐concentration conditions, celastrol could downregulate various forms of CHK1 through a specific mechanism. In contrast, the CHK1‐selective inhibitor rabusertib slightly downregulated CHK1 and pS296 CHK1, but upregulated pS345 CHK1. Additionally, we assessed the phosphorylation of histone H2AX as a biomarker of DNA damage by WB. Our findings revealed that both high concentrations of celastrol and rabusertib significantly induced DNA damage in Jurkat cells after 24 h of treatment (Figure 5F). Considering that CHK1 mediates the checkpoints during the S phase and M phase of the cell cycle, as well as the transition from G2 to M phase,^[^ ^27^ ^]^ we assessed the impact of celastrol on the cell cycle in Jurkat cells. The result indicated a significant G2/M phase arrest induced by celastrol, unlike the S phase arrest caused by rabusertib. This discrepancy might be attributed to the distinct mechanism and multi‐target nature of celastrol (Figure 5G). Inspired by the interaction mechanisms between celastrol and PI3Kα, as well as IKKβ, we initially speculated that celastrol might also interact with the kinase pocket of CHK1. Consequently, we conducted a homogeneous time‐resolved fluorescence (HTRF) assay to evaluate the kinase inhibitory activity of celastrol and the degrader ZH‐011 against CHK1. The result indicated that, in the presence of the positive compound AZD7762 (a CHK1 inhibitor) demonstrating pronounced inhibitory activity, neither celastrol nor ZH‐011 displayed significant inhibitory activity (Figure S9, Supporting Information). This finding aligned with the observed differences in the action mechanisms of celastrol and rabusertib in the CHK1 signaling pathway experiment.

Although celastrol did not exhibit CHK1 kinase inhibitory activity, a high concentration of celastrol could induce downregulation of the CHK1 protein level. Additionally, CHK1 could be effectively degraded and pulled down by ZH‐011. Therefore, we hypothesized that celastrol may act on other sites of CHK1 with a relatively weak binding affinity instead of direct binding to kinase domain, or it may modulate protein complexes in which CHK1 participates. This could explain why other strategies have failed to identify this target.

Overall, we speculated that CHK1 might be a potential toxicity target for celastrol, but further experiments are needed to substantiate this hypothesis.

### 2.6 OGA, CIP2A, and ERCC6L were Identified as the Potential Targets of Celastrol

OGA hydrolyzes the N‐acetylglucosamine moiety from proteins to regulate O‐GlcNAcylation, which plays a critical role in various human diseases such as cancer, diabetes, and neurodegeneration as a post‐translational modification. However, due to the lack of dynamic perturbation tools and chemical proteomic methods, the precise regulation of O‐GlcNAcylation in thousands of proteins within live cells and the monitoring of spatial‐temporal specificity or dynamic changes cannot be fully resolved.

**Figure 6:**
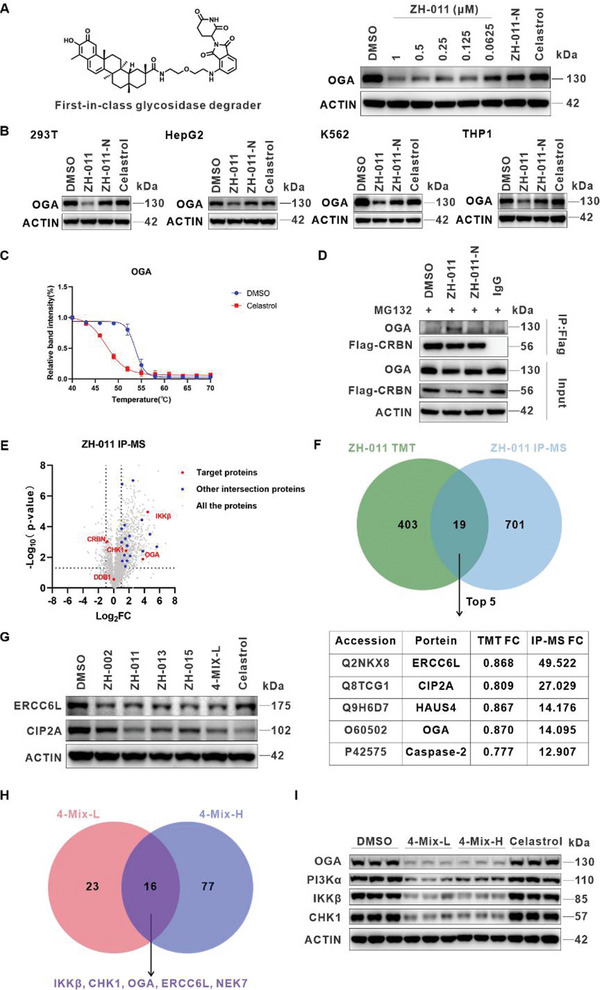
OGA, CIPA1, and ERCC6L were identified as the potential targets of celastrol. A) The dose‐dependent degradation experiment of OGA protein in Jurkat cell line treated with ZH‐011 for 8 h, the concentrations of ZH‐011‐N and celastrol were 500 nm. B) Degradation activity of ZH‐011 on OGA protein in 293T, HegpG2, THP1, and K562 cells. Cells were treated with DMSO, ZH‐011 (500 nm), ZH‐011‐N (500 nm), and celastrol (500 nm) for 24 h before WB. C) Melt and shift curve of OGA in Jurkat cells treated with DMSO control (blue) and 50 µm celastrol (red). Data are represented as mean ± SD, n = 3. D) Co‐immunoprecipitation of endogenous CHK1 from Flag‐CRBN in 293T cells after pretreatment with MG132 (5 µm) for 1 h, followed by treatment of DMSO, ZH‐011 (10 µm), ZH‐011‐N (10 µm) separately for 3 h. E) Flag immunoprecipitation followed by mass spectrometry in 293T cells overexpressing Flag‐CRBN of cells treated with MG132 (5 µm) alone or in combination with ZH‐011 (10 µm). Fold change and *p* value were calculated by comparing ZH‐011/MG132 treated samples with DMSO/MG132 treated samples. The significance analysis was conducted by two‐tailed unpaired Student's *t*‐tests using Microsoft Excel spreadsheets with basic statistical program (biological replicates; n = 2, *p* value <0.05). The red dots represented the target proteins we focused on, and the blue dots represented other proteins that intersected with the TMT data. F) The Venn diagram illustrated the intersection proteins of ZH‐011′s TMT data with IP‐MS data, and the table listed the top five proteins ranked by fold change in IP‐MS. G) Degradation activity of the PROTAC toolbox molecules on CIP2A and ERCC6L. Jurkat cells were treated with DMSO, ZH‐002 (500 nm), ZH‐011 (500 nm), ZH‐013 (500 nm), ZH‐015 (500 nm), 4‐Mix‐L (the total concentration of the four molecules was 500 nm) and celastrol (500 nm) for 8 h before WB. H) The Venn diagram showed the number of proteins downregulated specifically and commonly by the mixed toolbox molecules at two concentrations (the total concentrations of the four molecules were 500 nm and 1 µm). I) Four target proteins degradation efficacy with the mixed toolbox molecules treatment. Jurkat cells were treated with DMSO, celastrol (500 nm), and the mixed toolbox molecules at two different concentrations (500 nm, 1 µm) for 8 h before WB.

We first conducted a dose‐dependent experiment of the optimal molecule ZH‐011 on OGA. The WB result revealed that ZH‐011 exhibited potent degradation activity, with a DC_50_ value of 133 nm, marking it as the first reported glycosidase degrader (**Figure** **6**A). The degradation of OGA induced by ZH‐011 could be blocked in the CRBN^−/−^ Jurkat cells (Figure S6A, Supporting Information), providing evidence for the degradation mechanism of this molecule. Additionally, ZH‐011 maintained OGA degradation activity in many different cell lines (Figure 6B). Interestingly, in contrast to the first three proteins, the thermal stability of OGA protein was significantly reduced by celastrol, indicating a direct interaction between celastrol and OGA^[^ ^25a,b^ ^]^ (Figure 6C). Furthermore, OGA protein could be specifically pulled down by the optimal molecule ZH‐011 (Figure 6D).

To further confirm the result of Co‐IP experiment, we conducted an Immunoprecipitation‐Mass Spectrometry (IP‐MS) experiment with ZH‐011. We analyzed the data from two biological replicates together with the mean value (Extended Data S2, Supporting Information). While we successfully observed the bait proteins CRBN and DDB1, we were pleasantly surprised to find that the abundance of OGA protein detected in the ZH‐011 group was more than ten times that of the DMSO group. Additionally, we also observed CHK1 and IKKβ among the proteins significantly upregulated compared to the control group (Figure 6E). Subsequently, we compared the downregulated proteins in the ZH‐011 TMT‐labeled proteomics data (fold change <0.88, *p* value <0.05) with the upregulated proteins in the IP‐MS data (fold change >2, *p* value <0.05) and identified 19 overlapping proteins. These proteins were further sorted based on their fold change in the IP‐MS experiment, and we focused on validating the top two proteins, ERCC6L and CIP2A (Figure 6F).

CIP2A is an oncogenic protein that regulates the phosphorylation of Akt and stabilizes c‐Myc. Previous studies have shown that celastrol can directly bind to CIP2A and promote the interaction between CIP2A and the E3 ligase CHIP, leading to the ubiquitination and degradation of CIP2A via a molecular glue mechanism.^[^ ^28^ ^]^ The WB result demonstrated that celastrol could significantly induce the degradation of CIP2A in Jurkat cells. However, PROTAC molecules designed based on celastrol have a larger molecular scaffold, resulting in reduced degradation activity on CIP2A (Figure 6G). Furthermore, IP‐MS data indicated that, apart from CIP2A, the E3 ligase CHIP could also be significantly pulled down by ZH‐011 (Extended Data S2, Supporting Information). In conclusion, our results robustly substantiated the findings of previous studies.

ERCC6L is a recently discovered DNA helicase that is highly expressed in various cancer cells, promoting cell cycle progression and stimulating cancer cell proliferation. The WB result showed that all the PROTAC toolbox molecules could lead to the downregulation of ERCC6L, whereas celastrol could not. This result suggested that ERCC6L might be another toxic target of celastrol similar to CHK1 (Figure 6G). We believed that combining quantitative proteomics with IP‐MS can effectively identify the target proteins that both form ternary complexes and undergo degradation when induced by PROTAC molecules, thereby eliminating interference from false‐positive proteins.

In conclusion, through the integrated analysis of TMT‐labeled proteomics data with IP‐MS data, we not only validated previously identified targets IKKβ, CHK1, and OGA, but also discovered another known target of celastrol, CIP2A, and a potential new target, ERCC6L. Furthermore, we developed the first glycosidase degrader capable of modulating intracellular O‐GlcNAcylation levels, which is expected to facilitate the study of this important post‐translational modification process.

### 2.7 Mixed‐Toolbox Quantitative Proteomics

In the aforementioned experiments, we employed single‐compound quantitative proteomics similar to the ABPP strategy. Building on this, we further introduced a mixed‐toolbox quantitative proteomics method to enhance the efficiency and cost‐effectiveness of target identification. To demonstrate the feasibility of this concept, we evenly mixed the celastrol‐based PROTAC toolbox and treated Jurkat cells for 8 h at different concentrations (4‐Mix‐L and 4‐Mix‐H, representing total concentrations of the four molecules were 500 nm and 1 µm, respectively), followed by quantitative proteomics experiments. Encouragingly, utilizing this optimized strategy, we successfully identified IKKβ, CHK1, OGA, ERCC6L, and NEK7 once again. However, PI3Kα and DCAF16 were not detected, possibly due to their low expression in Jurkat cells (Figure 6H). Additionally, we validated the degradation of IKKβ, PI3Kα, CHK1, and OGA induced by the mixed toolbox using WB (Figure 6I). Our results revealed that the mixed toolbox exhibited comparable or even superior degradation activity for several target proteins, including IKKβ, PI3Kα, CHK1, OGA, CIP2A, ERCC6L, suggesting its potential in the DBPP strategy. We firmly believed that this refinement will significantly enhance the efficiency and reduce the cost of target identification, ultimately leading to a substantial acceleration in the development of both natural products and other drug molecules.

## 3 Summary and Prospect

In this study, we combined the emerging PROTAC technology with quantitative proteomics to develop a novel DBPP strategy based on a diverse PROTAC toolbox for efficient target identification of natural products. We applied this strategy to celastrol and successfully identified multiple potential targets, including IKKβ, PI3Kα, CHK1, OGA, CIP2A, ERCC6L, NR4A1, DCAF16, and NEK7, demonstrating the potent capability of the DBPP strategy. To confirm these targets, we employed various biological techniques, including WB, Co‐IP, CETSA, and IP‐MS. Especially, through the comparison of TMT‐labeled proteomics data with the IP‐MS data, we were not only able to validate previously identified targets such as IKKβ, CHK1, and OGA, but also accurately discovered additional potential targets, including ERCC6L and CIP2A. One of the most noteworthy achievements of this study was the development of the first glycosidase degrader, expanding the scope of PROTAC applications to modulate enzymes involved in crucial post‐translational modification processes. In addition, the improved mixed‐toolbox quantitative proteomics method demonstrated reduced costs and increased efficiency, further amplifying the potential of this new strategy.

The DBPP strategy is not limited to target identification of natural products but can also be applied to study off‐target effects of existing drugs and repurpose old drugs for new uses. For natural products or drug molecules with multiple modification sites, researchers should design a diverse PROTAC toolbox with multiple sites, types, and lengths, and choose single‐toolbox or mixed‐toolbox quantitative proteomics based on practical considerations. To minimize the impact of chemical modifications on the compound's binding affinity, we can conduct appropriate phenotype screening based on the compound's pharmacological activity to select the most suitable PROTAC toolbox for target identification. We are confident that the application of mixed‐toolbox IP‐MS in subsequent studies will further expedite target identification. It is worth emphasizing that the comprehensive analysis of quantitative proteomics data and IP‐MS data will provide a fresh perspective on target identification and reduce false‐positive results. Through this method, we can identify target proteins that can both form ternary complexes and undergo degradation, those that can form ternary complexes but cannot undergo degradation, and those that cannot form ternary complexes but can undergo degradation. Although we solely employed CRBN as the E3 ligase in this study for proof of concept, we believe that in future DBPP strategies, a more diverse range of E3 ligases, such as VHL, should be considered.

In conclusion, we firmly believe that our innovative DBPP strategy can complement existing chemical proteomics technologies and greatly advance the field of pharmaceuticals. By facilitating efficient target identification, this strategy has the potential to accelerate drug discovery, ultimately uncovering novel therapeutic targets and fostering the development of more potent drugs. Moreover, further research and validation of these identified targets are necessary to provide valuable insights into the mechanisms of action of celastrol and open new avenues for therapeutic interventions in various diseases in the future.

## 4 Experimental Section

### Chemical Materials, Methods, Synthetic Route for PROTAC Compounds Library

The details of materials, methods, synthetic route are described in Supporting Information (Chemistry Part).

### Cell Line Culturing

All cell lines were tested for mycoplasma contamination according to the manufacturer's guide (Mycoblue Mycoplasma Detector, D101‐01). 293T cell line was obtained from ATCC. The Jurkat cell line was kindly provided by Dr. Li Haitao from the School of Life Sciences (Tsinghua University, Beijing, China). The HepG2 cell line was kindly provided by Dr. He Fuchu from the National Center for Protein Sciences (Beijing).

293T, Flag‐CRBN 293T, Flag‐DCAF16 293T and HepG2 cells were maintained in DMEM (Gibco) supplemented with 10% FBS (BI) and 1% Penicillin‐Streptomycin. Jurkat cells were maintained in RPMI 1640 (Gibco) supplemented with 10% FBS (BI) and 1% Penicillin‐Streptomycin. All cell lines were cultured at 37 °C with 5% CO_2_.

### Western Blot and Protein Degradation Assay

Cells were cultured under different compound treatment conditions. Cells were seeded in 6 well or 12 well plates. After treatment, cells were collected and washed with PBS, then lysed in 5X loading buffer (Yeasen, 20315ES) containing mercaptoethanol. Then, samples were heated in a 100 °C metal bath for 10 min. Cell lysates were added to 8–12% SDS‐PAGE gels and transferred to the PVDF membrane (Merck Millipore). After transfer, the membrane was blocked with 5% non‐fat milk in TBST for 1 h at room temperature and shaking. The membrane was incubated with 1:1000 anti‐IKKβ (Abcam, ab124957), anti‐PI3K p110α (CST, 4249S), anti‐PI3K p110β (CST, 3011S), anti‐PI3K p110γ (CST, 5405S), anti‐CHK1 (Abcam, ab32531), anti‐Flag (Abmart, M20008), anti‐NEK7 (Abcam, ab133514), anti‐OGA (Abcam, ab124807), anti‐CIP2A (Abcam, ab99518), anti‐PICH (CST, 8886S), anti‐NUR77 (Abcam, ab283264), and anti‐β‐Actin (ABclonal, AC026) antibody diluted in the corresponding antibody dilution buffer (NCM Biotech, WB500D) and incubated overnight at 4 °C, with shaking. Then, the membrane was incubated with 1:5000 appropriate HRP‐conjugated secondary antibodies for 1 h at RT and washed five times in TBST for 5 min. Membranes were developed with enhanced chemiluminescence (NCM Biotech, P2200) and imaged using a Tanon 5200 luminous. The grayscale analysis was conducted by ImageJ software. Statistical analysis was carried out using GraphPad Prism v.8.

### Cloning

Human CRBN complementary DNA with N terminal Flag tag was obtained by PCR with reverse transcription (RT‐PCR) amplification of a cDNA pool extracted from 293T cells and subcloned via Gibson Assembly (Vazyme, C113‐01) into pLenti‐EF1α‐IRES‐mCherry vector. Human DCAF16 cDNA with N terminal Flag tag was obtained by RT‐PCR amplification of a cDNA pool extracted from 293T cells and subcloned via Gibson Assembly (Vazyme, C113‐01) into pLenti‐EF1α‐IRES‐mCherry vector.

### Generation of Flag‐CRBN and Flag‐DCAF16 stably Expressed 293T Cell Lines by Lentivirus Transduction

Flag‐CRBN or Flag‐DCAF16 lentivirus was generated by co‐transfection of Flag‐CRBN or Flag‐DCAF16, psPAX2 and pMD2.G into 293T cells using Lipofectamine 3000 (L3000015). Virus‐containing medium were collected 48 h after transfection, filtered with a 0.45 µm filter and used to transduce 293T cells. Then, 72 h after transduction, cells were sorted by fluorescent‐activated cell sorting for mCherry using MoFlo Astrios EQ flow sorter.

### Cellular Thermal Shift Assay

CETSA experiments were carried out as described in previous resports.^[^ ^29^ ^]^ Jurkat cells were harvested and washed with PBS. The cells were diluted in Kinase buffer (CST, 9802S) for PI3Kα, CHK1 and IKKβ. All buffers were supplemented with protease inhibitor (Yeasen, 20123ES10). DMSO and 50 µm celastrol were added and incubated at 37 °C for 1 h. Each tube of cells was then aliquoted into PCR tubes, and heated at 70, 67, 64, 61, 58, 55, 52, 49, 46, 43, and 40 °C for 3 min in 96‐well thermal cycler. Cells were freeze‐thaw cycled three times and cell lysates were centrifuged at 20 000 g for 20 min at 4 °C. The supernatant was carefully transferred and analyzed by WB after mixing with 5×loading buffer and boiling.

### Co‐Immunoprecipitation

Flag‐CRBN 293T were seeded in 6‐well plates. After 24 h, cells were treated with 5 µm MG132 (TargetMol, T2154) for 1 h. PROTAC molecule was added to a final concentration of 10, 5, and 1 µm and the cells were incubated at 37 °C for 2 h. Cells were collected and lysed in IP lysis buffer (Beyotime, P0013) with protease inhibitor on ice for 30 min, followed by centrifugation at 13 400 g for 10 min collection of the supernatant. Cell lysates were incubated with anti‐Flag magnetic beads (Beyotime, P2115) at 4 °C for overnight and washed three times with washing buffer. The beads were diluted with TBS (Beyotime, ST661), mixed with 2×SDS loading buffer and heated at 95 °C for 5 min, followed by WB analysis.

### Immunoprecipitation Mass Spectrometry (IP‐MS)

For IP‐MS experiments, immunoprecipitation (IP) was performed as described above. Then substrate was separated by SDS‐PAGE, and subjected to Coomassie staining. Desired bands corresponding to different molecular weights are excised from the gel and subjected to in‐gel digestion with trypsin. Each excised band is carefully chopped into 1 mm cubes, placed in 1.5 mL Eppendorf tube containing 50% acetonitrile with 25 mm ammonium bicarbonate, pH 8.0, and incubated at 37 °C for 30 min to destain. Samples are reduced with 5 mm DTT for 30 min at 45 °C and alkylated with 10 mm iodoacetamide for 30 min at room temperature in the dark. The dehydrated gel pieces are digested with trypsin (Trypsin Gold /protein = 1/50) overnight at 37 °C.

The peptides are extracted twice with 50% acetonitrile and 0.1% trifluoroacetic acid, each for 45 min at 37 °C; the extracts are combined. The extracts are evaporated under vacuum and resuspended in 25 µL of H_2_O (0.1% FA) for LC‐MS/MS analysis.

For LC‐MS/MS analysis, the peptides were separated by a 120 min gradient elution at a flow rate 0.250 µL min^−1^ with a Thermo‐Dionex Ultimate 3000 HPLC system, which was directly interfaced with a Thermo Scientific Q Exactive HFX mass spectrometer. The analytical column was a home‐made fused silica capillary column (75 µm ID, 150 mm length; Upchurch, Oak Harbor, WA) packed with C‐18 resin (300 Å, 5 µm, Varian, Lexington, MA). Mobile phase A consisted of 0.1% formic acid, and mobile phase B consisted of 100% acetonitrile and 0.1% formic acid. The QEHFX mass spectrometer was operated in the data‐dependent acquisition mode using Xcalibur 4.4 software and there was a single full‐scan mass spectrum in the orbitrap (400‐1800 m/z, 120 000 resolution) followed by top‐speed MS/MS scans at 27% normalized collision energy (HCD).

### LC‐MS Data Analysis for IP‐MS

The MS/MS spectra from each LC‐MS/MS run were searched against the human.fasta from UniProt (release date of Feb.6, 2023) using an commercial Proteome Discoverer (Version PD 3.0, Thermo‐Fisher Scientific, USA). The search criteria were as follows: full tryptic specificity was required; two missed cleavage was allowed; carbamidomethylation (C) were set as the fixed modifications; the oxidation (M), Acetyl (N‐terminal), Met‐loss (N‐terminal) and Acetyl+Met‐loss (N‐terminal) were set as the variable modification; precursor ion mass tolerances were set at 10 ppm for all MS acquired in an orbitrap mass analyzer; and the fragment ion mass tolerance was set at 20 mmu for all MS2 spectra acquired. The peptide false discovery rate (FDR) was calculated using Percolator provided by PD. When the q value was smaller than 1%, the peptide spectrum match (PSM) was considered to be correct. FDR was determined based on PSMs when searched against the reverse, decoy database. Peptides only assigned to a given protein group were considered as unique. The false discovery rate (FDR) was also set to 0.01 for protein identifications

### Cell Proliferation and Cell Cycle Analyses

To assess the effect of compounds on proliferation, cells (10 000 100 µL^−1^) were seeded in 96‐well plates followed by addition of compound at indicated concentrations. After 72 h, 100 µL per well of reconstituted CCK‐8 reagent (Yeasen, 40203ES76) was added and read on a microplate reader from BioTek. The absorbance indicated the survival rate. Survival rate could be calculated using the formula below:

(1) $$\begin{array}{ccc} & & \mathrm{Relative}\;\mathrm{growth}\;\mathrm{rate}\left(\%\right)\\ & & \;=\frac{\mathrm{Drug}\;\;\mathrm{absorption}\;\mathrm{value}-\mathrm{Blank}\;\;\mathrm{absorption}\;\mathrm{value}}{\mathrm{DMSO}\;\;\mathrm{absorption}\;\mathrm{value}-\mathrm{Blank}\;\;\mathrm{absorption}\;\mathrm{value}}\\ & & \;\times 100 \end{array}$$

For each concentration, at least three replica wells were examined. Relative growth rate curves were determined by nonlinear regression curve fit using GraphPad Prism v.8.

Cell cycle was determined using DNA content quantitation assay (Beijing Solarbio Science & Technology, CA1510), according to manufacturer's protocol. Finally, the labeled cells were analyzed by FlowJo software. For each concentration of every compound, at least three replica wells were assessed. The bar chart was plotted using GraphPad Prism v.8.

### TMT‐Labeled Quantitative Proteomics Assay

Quantitative proteomics were performed using Deng's method.^[^ ^30^ ^]^ To obtain TMT‐labeled quantitative proteomics data, Jurkat cells were first transplanted into 6 cm petri dishes. Then, compounds of interest and blank control DMSO were added to the culture medium, respectively, and treated for 8 h. For each group, three biological replicates were set. Then, the cells were collected by centrifuge at 4 °C, 300 g. Cells were than washed twice with cold PBS, and lysed with ultrasonication in 8m urea at 4 °C for 6 min, followed by centrifugation at 12 000 g and the supernatant was collected and quantified (using a Solarbio BCA kit, PC0020), followed by digestion and TMT labeling. The resulting spectra from each run were searched separately against uniprot human database by the search engines: Proteome Discoverer (PD, Thermo, HFX and 480). The protein quantitation results were statistically analyzed by T‐test. The proteins whose quantitation significantly different between experimental and control groups (FC <0.88 and *p* value <0.05) were defined as significantly expressed proteins. The scatter diagram was plotted using GraphPad Prism v.8.

### In Vitro Kinase Assays

HTRF assays were conducted by MedChemExpress (MCE). Add 50 µL compound to 384 well dilution plate, then dilute compound 1:3/1:4 in succession in DMSO for each column for ten different concentrations. Transfer 0.05 µL diluted compound solution in each row to 384 assay plate using Echo, each column containing 2 replicates. Add 2.5 µL enzyme working solution to 384 well assay plate, centrifuge 1000 rpm for 1 min and incubate at 25 °C for 10 min. Add 2.5 µL substrate (ATP & STK S1) working solution to initiate reaction and incubate at 25 °C for 60 min. Add 5 µL substrate (STK Ab&XL 665) working solution to initiate reaction and incubate at 25°C for 60 min. Finally, Reading 620 and 665 nm fluorescence signals with BMG (Ratio 665/620).

(2) $$\begin{array}{ccc} & & \mathrm{Percent}\;\mathrm{inhibition}\left(\%\right)\\ & & \;=\frac{\mathrm{ave}\;\;\mathrm{DMSO}\;\;\mathrm{and}\;\;\mathrm{enzyme}-\mathrm{compound}\;\mathrm{well}\;}{\mathrm{ave}\;\;\mathrm{DMSO}\;\;\mathrm{and}\;\;\mathrm{enzyme}\;-\mathrm{ave}\;\;\mathrm{DMSO}\;\;\mathrm{and}\;\;\mathrm{assay}\;\mathrm{buffer}}\\ & & \;\times 100 \end{array}$$

Fit the compound IC_50_ from nonlinear regression equation by XLfit 5.5.0.

### Gene Ontology (GO) Analysis

Gene Ontology (GO) analysis was conducted using R package “clusterProfiler^[^ ^31^ ^]^” to find biological pathway with Benjamini and Hochberg methods to adjust *p* value and threshold was set to 0.05.

### Statistical Analysis

Most experiments were conducted with three biological replicates. The data from multiple experiments were analyzed using GraphPad Prism v.8 and are presented as the mean ± standard deviation (SD). The significance analysis was conducted by two‐tailed unpaired Student's *t*‐tests using Microsoft Excel spreadsheets with basic statistical program. When *p* value <0.05, it was considered that there were significant differences between the groups.

### Data Availability

The mass spectrometry proteomics data have been deposited to the ProteomeXchange Consortium (http://proteomecentral.proteomexchange.org) via the iProX partner repository^[^ ^32^ ^]^ with the dataset identifier PXD044955.

## Conflict of Interest

The authors declare no conflict of interest.

## Author Contributions

Z.N. and Y.S. contributed equally to this work. Y.R. designed the project. Y.R., Z.N., and Y.S. designed the experiments, analyzed the data, and wrote the manuscript. Z.N. designed and synthesized the molecules. Z.N. and Y.S. performed WB experiments. Y.S. performed cell proliferation experiments, cell cycle experiments, Co‐IP experiments, CETSA experiments and constructed the Flag‐CRBN cell line. L.W. performed molecular dynamics stimulation. Q.L. performed GO analysis. X.S. provided support for mass spectrum experiments.

## Supporting information

Supporting Information

Supporting Information

Supporting Information

Supporting Information

Supporting Information

## Data Availability

The data that support the findings of this study are available in the supplementary material of this article.
