# Supplementary material for: Degradation‐Based Protein Profiling: A Case Study of Celastrol
Source: Adv Sci (Weinh). 2024 Apr 25;11(25):2308186. doi: 10.1002/advs.202308186 (PMC11220716; doi:10.1002/advs.202308186)

ADVANCED SCIENCE
Open Access

Supporting Information

for *Adv. Sci.*, DOI 10.1002/advs.202308186

Degradation-Based Protein Profiling: A Case Study of Celastrol

*Zhihao Ni, Yi Shi, Qianlong Liu, Liguo Wang, Xiuyun Sun and Yu Rao**

Degradation-Based Protein Profiling: A Case Study of Celastrol

Zhihao Ni, Yi Shi, Qianlong Liu, Liguo Wang, Xiuyun Sun, Yu Rao[*]

Z.Ni, Y. Shi, Q. Liu, L. Wang, Y. Rao

MOE Key Laboratory of Protein Sciences, School of Pharmaceutical Sciences, MOE Key Laboratory of Bioorganic Phosphorus Chemistry & Chemical Biology, Tsinghua University, Beijing, China.

E-mail: yrao@tsinghua.edu.cn

X. Sun, Y. Rao

Changping Laboratory, 102206 Beijing, China.

Supplementary Information (Biology Part)

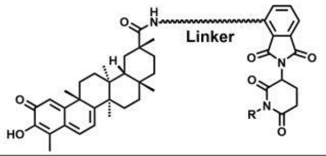

ID	Linker	R	MW
Celastrol			450.28
ZH-001	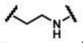	H	748.38
ZH-002	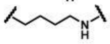	H	776.41
ZH-002-N	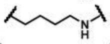	Et	804.45
ZH-003	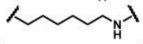	H	804.45
ZH-004	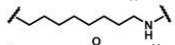	H	832.48
ZH-005	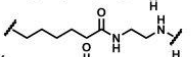	H	861.47
ZH-006	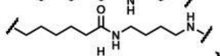	H	889.50
ZH-007	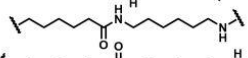	H	917.53
ZH-008	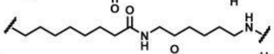	H	945.56
ZH-009	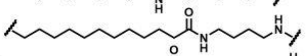	H	973.59
ZH-010	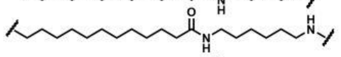	H	1001.62
ZH-011	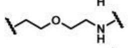	H	792.41
ZH-011-N	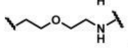	Et	820.44
ZH-012	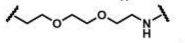	H	836.44
ZH-013	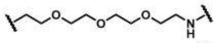	H	880.46
ZH-013-N	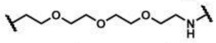	Et	908.49
ZH-014	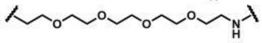	H	924.49
ZH-015	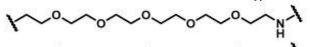	H	968.51
ZH-015-N	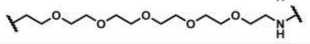	Et	996.55

Table S1. The PROTAC library based on celastrol.

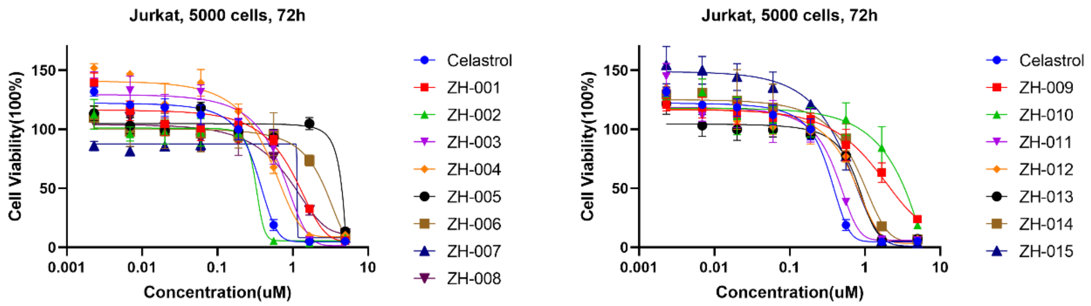

Figure S1. In-vitro proliferation inhibition in Jurkat cells of PROTAC library based on celastrol. In 96-well plates, 5000 cells were incubated in each well at 37°C for 72 h. The final result was indicated by CCK-8. Data shown are representative of three independent experiments. Data are represented as mean ± SD, n = 3.

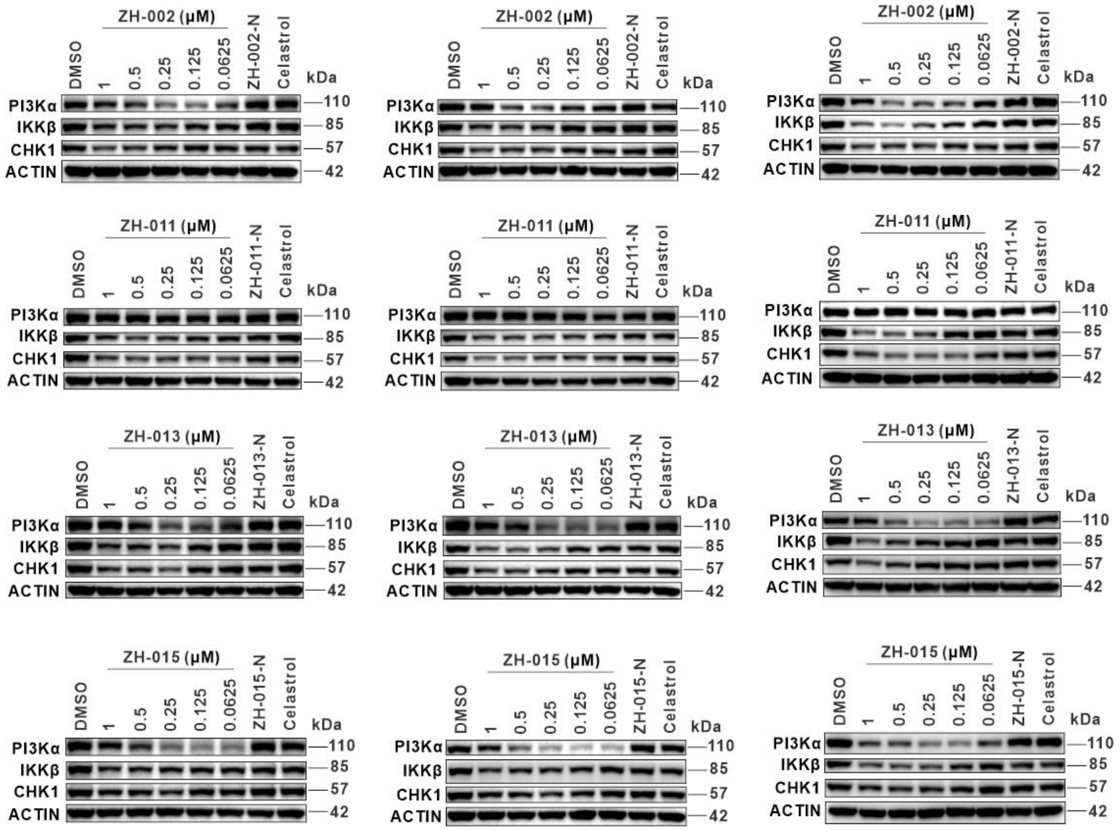

Figure S2. Immunoblot for PI3Kα, IKKβ, CHK1 and β-actin in Jurkat cells treated with the toolbox molecules. Jurkat cells were incubated with indicated concentration of ZH-002, ZH-011, ZH-013, ZH-015, ZH-002-N (500 nM), ZH-011-N (500 nM), ZH-013-N (500 nM), ZH-015-N (500 nM), celastrol (500 nM) for 8 h before lysis and western blot analysis. The gels are representative of three biologically independent experiments.

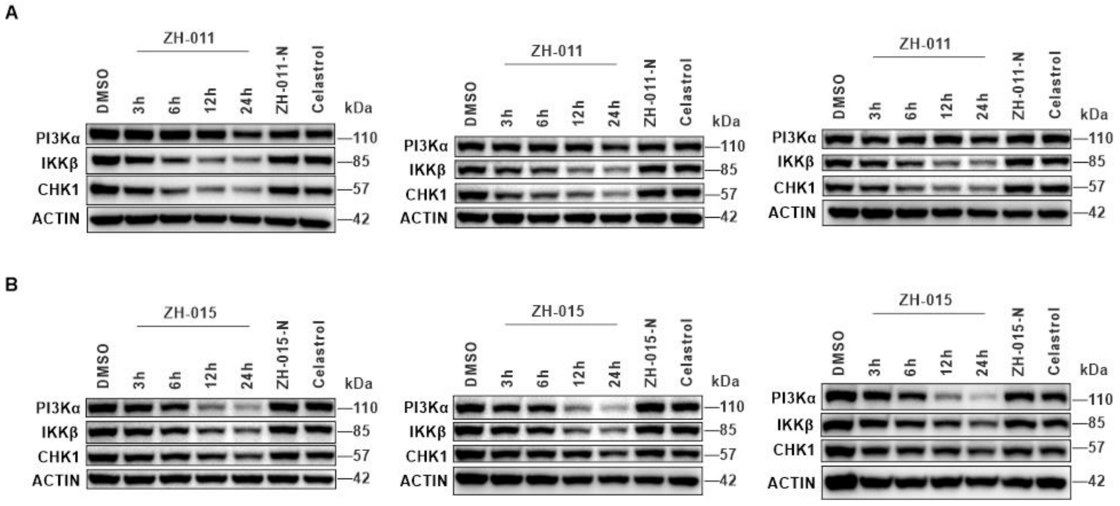

Figure S3. The time dependent degradation of PI3Kα, IKKβ and CHK1 in Jurkat cells treated with ZH-011 or ZH-015. Jurkat cells were incubated with ZH-011 (500 nM) or ZH-015 (500 nM) for 3, 6, 12, and 24 h, respectively, and incubated with ZH-011-N (500 nM), ZH-015-N (500 nM) and celastrol (500 nM) for 24 h before lysis and western blot analysis. The gels are representative of three biologically independent experiments.

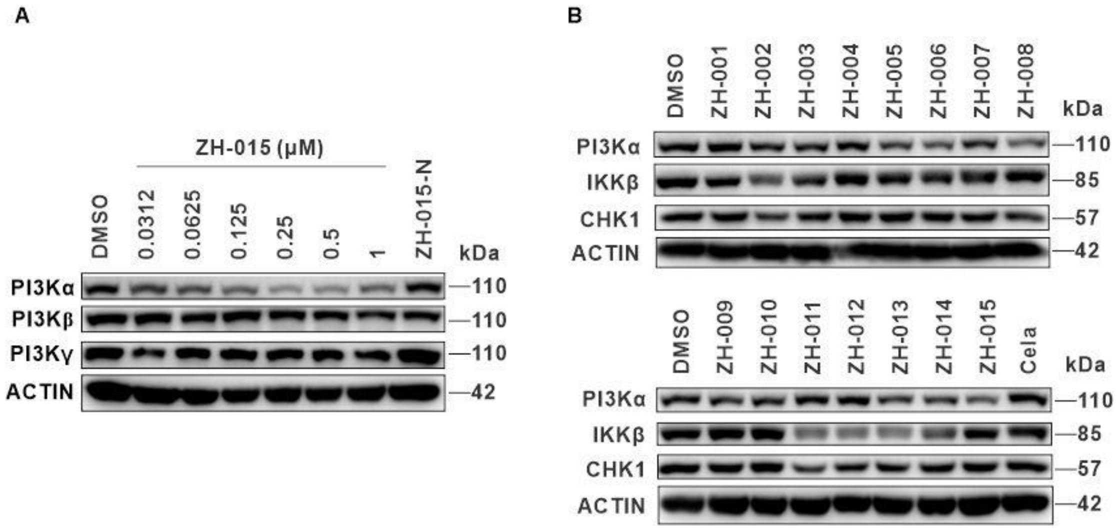

Figure S4. (A) Immunoblot for PI3Kα, PI3Kβ, PI3Kγ and β-actin in Jurkat cells treated with the ZH-015. Jurkat cells were incubated with indicated concentration of ZH-015 and ZH-015-N (500 nM) for 8 h before lysis and western blot analysis. (B) Immunoblot for PI3Kα, IKKβ, CHK1 and β-actin in Jurkat cells treated with the entire compound library at a concentration of 500 nM for 8 h.

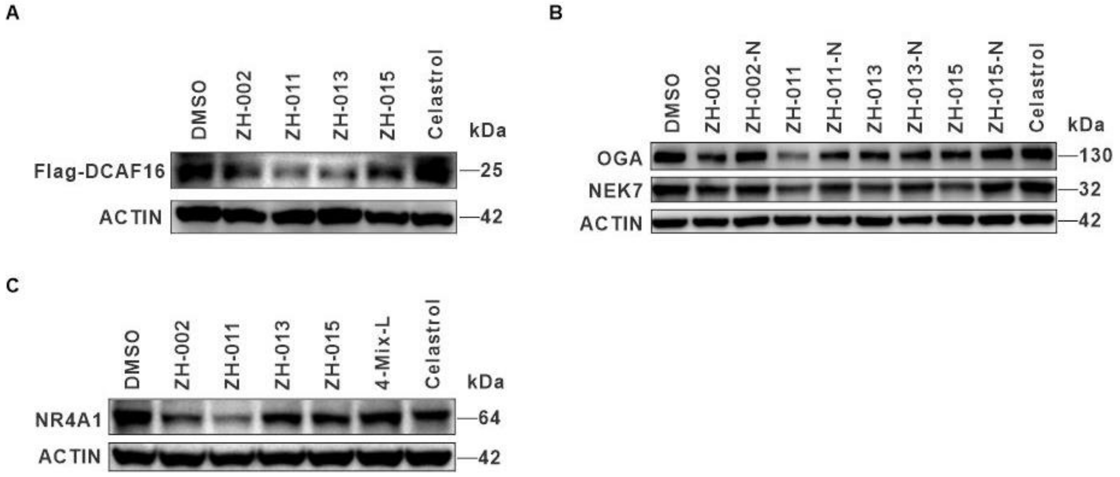

Figure S5. (A) Immunoblot for Flag-DCAF16 and β-actin in Flag-DCAF16 293T cells treated with 500 nM ZH-002, ZH-011, ZH-013, ZH-015 and celastrol for 8 h. (B) Immunoblot for OGA, NEK7 and β-actin in Jurkat cells treated with 500 nM ZH-002, ZH-002-N, ZH-011, ZH-011-N, ZH-013, ZH-013-N, ZH-015, ZH-015-N and celastrol for 8 h. (C) Immunoblot for NR4A1 and β-actin in Hela cells treated with 500 nM ZH-002, ZH-011, ZH-013, ZH-015, 4-Mix-L (the total concentration of the four molecules was 500 nM) and celastrol for 8 h.

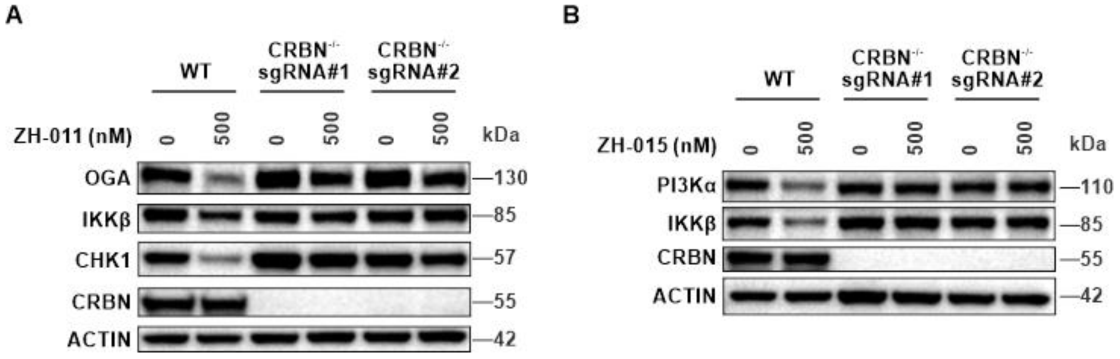

Figure S6. (A) Immunoblot for OGA, IKKβ, CHK1, CRBN and β-actin in wild-type and CRBN-KO (CRBN-/-) Jurkat cells treated with 500 nM ZH-011 for 8 h. (B) Immunoblot for PI3Kα, IKKβ, CRBN and β-actin in wild-type and CRBN-KO (CRBN-/-) Jurkat cells treated with 500 nM ZH-015 for 8 h.

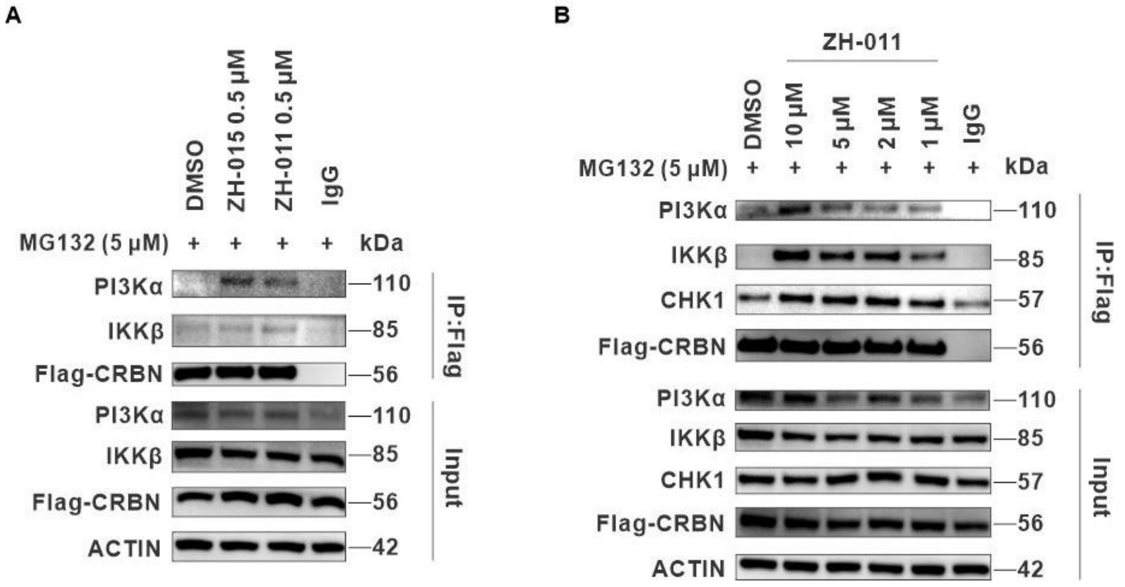

Figure S7. (A) Co-immunoprecipitation of endogenous PI3Kα and IKKβ from Flag CRBN in 293T cells after pretreatment with MG132 (5 μM) for 1 h, followed by treatment of DMSO, ZH-011 (500 nM), ZH-015 (500 nM) separately for 3 h. (B) Co-immunoprecipitation of endogenous PI3Kα, IKKβ and CHK1 from Flag-CRBN in 293T cells after pretreatment with MG132 (5 μM) for 1 h, followed by treatment of DMSO, ZH-011 (1 μM, 2 μM, 5 μM, 10 μM) separately for 3 h.

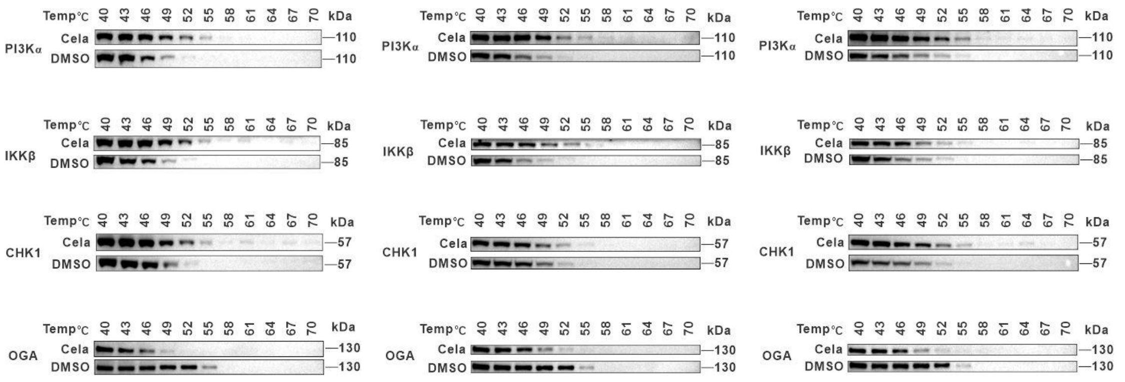

Figure S8. Thermal stability of PI3Kα, IKKβ, CHK1 and OGA affected by celastrol (50 μM) in live cells was measured by temperature-dependent CETSA before western blot analysis. The data are from three independent experiment.

A

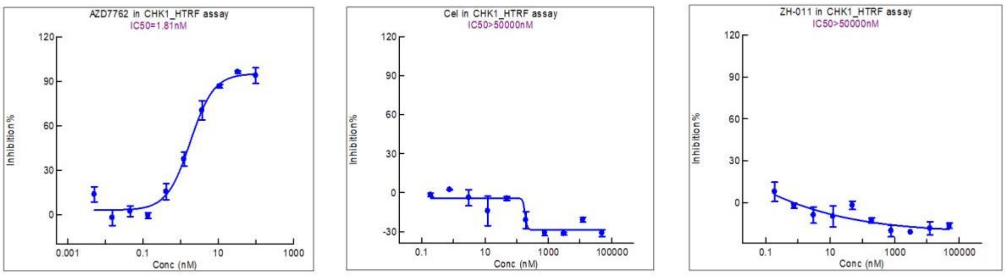

B

Assay name	Compounds ID	IC_{50} (nM)
CHK1 HTRF assay	AZD7762	1.81
	Celastrol	> 50000
	ZH-011	> 50000

Figure S9. (A) Enzymatic inhibition curves of AZD7762, celastrol, and ZH-011 against CHK1. (B) The enzymatic IC_{50} values of AZD7762, celastrol, and ZH-011 on CHK1. All the IC_{50} values were tested in duplicate, and the raw data is presented in Extended Data 4. Enzymatic inhibition curves were determined by nonlinear regression curve fit using XLfit 5.5.0.

Supplementary Information (Chemistry Part)

Materials and methods

All commercial materials (Adamas-beta, Bidepharm, Energy Chemical, Aladdin etc.) were used without further purification. All solvents were analytical grade. MG132 (MCE, HY-13259, 133407-82-6), MLN4924 (TargetMol, T6332, 905579-51-3), carfilzomib (Aladdin, C127870, 868540-17-4), rabusertib (MCE, HY-14720, 911222-45-2) were purchased. The ^{1}H NMR and ^{13}C NMR spectras were recorded on a Bruker AVANCE III 400 MHz spectrometer in $CDCl_3$ using solvent peak as a standard. All ^{13}C NMR spectras were recorded with complete proton decoupling. Low-resolution mass spectral analysis was performed with an Agilent 6340 or Waters AQUITY UPLCTM/MS. Analytical TLC was performed on Yantai Chemical Industry Research Institute silica gel 60 F254 plates and flash column chromatography was performed on Qingdao Haiyang Chemical Co. Ltd silica gel 60 (200-300 mesh). The rotavapor was BUCHI’s Rotavapor R-3.

Synthetic route for PROTACs compounds library

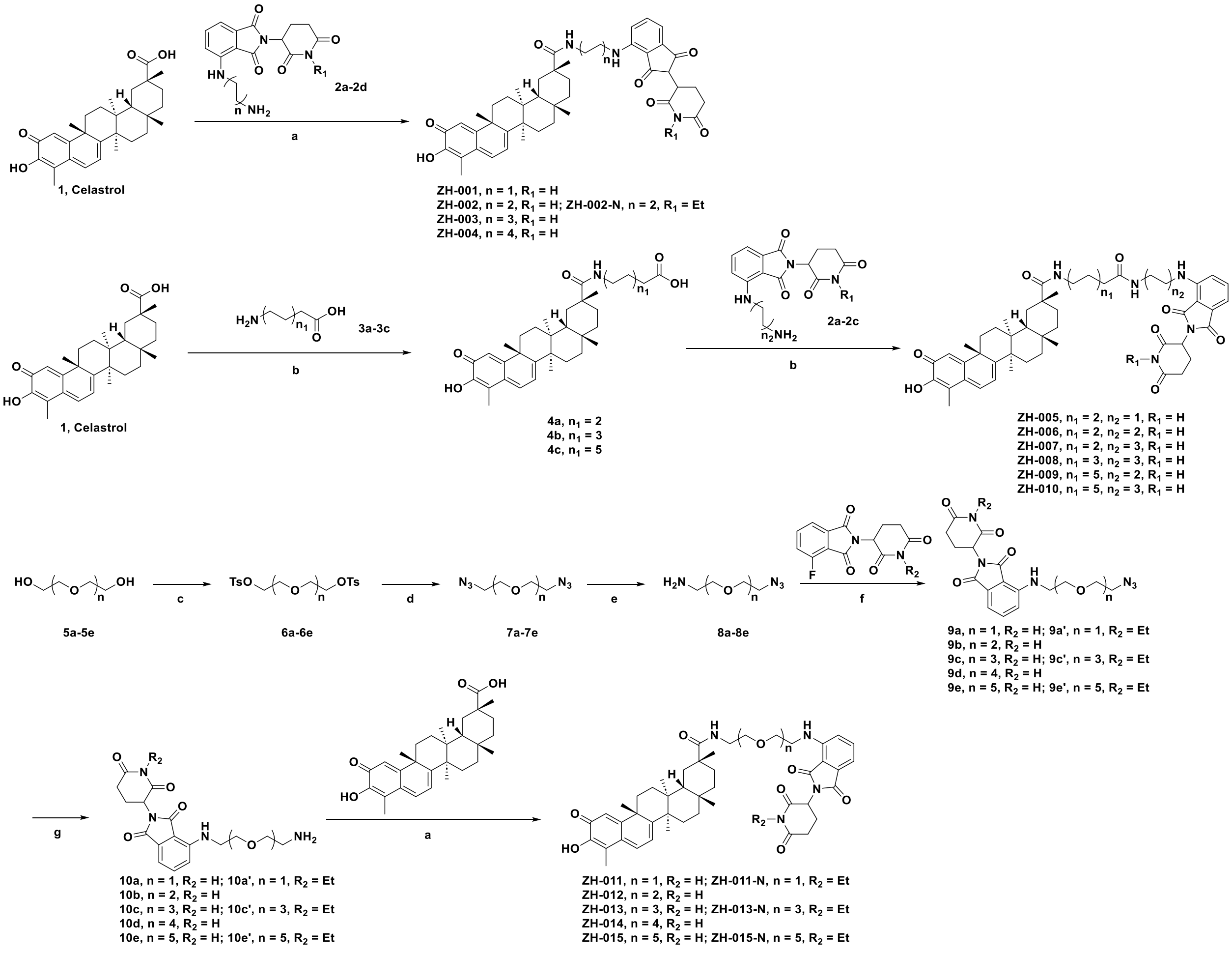

Condition: a) HATU, DIPEA, DCM, rt, 3 h; b) HATU, DIPEA, DMF, 60°C, 3 h; c) TsCl, Et_3N, DCM, rt, overnight; d) TMS-N_3, TBAF, THF, 60°C, 2 h; e) Pph_3, 4M HCl, PhMe, rt, overnight; f) DIPEA, DMSO, 85°C, 4 h; g) Pd/C, H_2, THF, rt, 12 h;

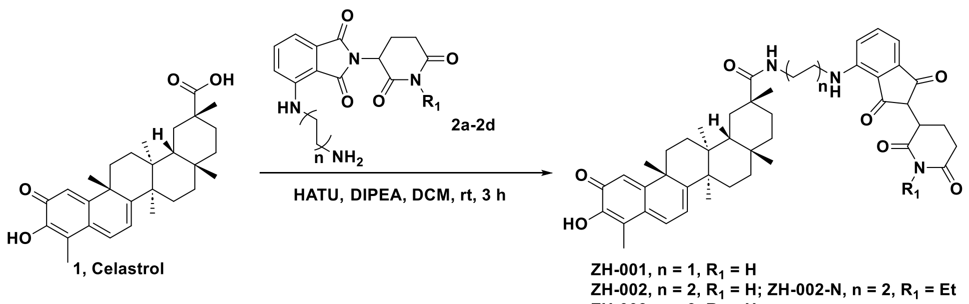

ZH-001, n = 1, R_1 = H
ZH-002, n = 2, R_1 = H; ZH-002-N, n = 2, R_1 = Et
ZH-003, n = 3, R_1 = H
ZH-004, n = 4, R_1 = H

Celastrol (13.5 mg, 0.03 mmol), HATU (12.54 mg, 0.033 mmol, 1.1 eq.), DIPEA (15 µL, 0.09 mmol, 3 eq.) were dissolved in DCM and stirred at room temperature for 5 min. Then the compounds 2a-2d (0.03 mmol, 1 eq.) in DCM were added respectively. The mixture was stirred for another 3 h. The resulting mixture was poured into water and extracted with DCM, the organic layer was concentrated and further purified by silica gel column chromatography (DCM: MeOH = 50: 1 to

DCM: MeOH = 30: 1) to give compounds ZH-001, ZH-002, ZH-002-N, ZH-003, ZH-004, IY = 50%.

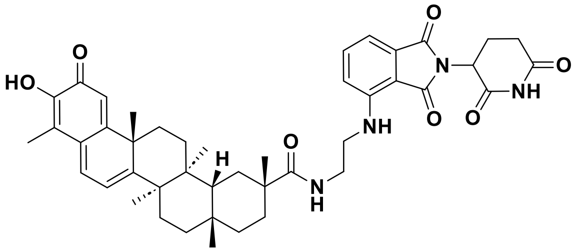

(2R,4aS,6aR,12bR,14aS,14bR)-N-(2-((2-(2,6-dioxopiperidin-3-yl)-1,3-dioxoisoindolin-4-yl)amino)ethyl)-10-hydroxy-2,4a,6a,9,12b,14a-hexamethyl-11-oxo-1,2,3,4,4a,5,6,6a,11,12b,13,14,14a,14b-tetradecahydropicene-2-carboxamide (ZH-001). ^{1}H-NMR (400 MHz, $CDCl_3$) δ 8.23 (d, J = 16.24 Hz, 1H), 7.38-7.29 (m, 1H), 6.99-6.92 (m, 2H), 6.85 (dd, J_1 = 18.76 Hz, J_2 = 8.4 Hz, 1H), 6.46 (d, J = 8.72 Hz, 1H), 6.29-6.24 (m, 2H), 6.13 (s, 1H), 4.94-4.89 (m, 1H), 3.46-3.36 (m, 4H), 2.95-2.74 (m, 3H), 2.44 (d, J = 15.52 Hz, 1H), 2.23 (d, J = 4.92 Hz, 3H), 2.20-1.25 (m, 17H), 1.21 (d, J = 4.92 Hz, 3H), 1.15 (s, 3H), 1.10 (s, 3H), 1.02-0.99 (m, 1H), 0.50 (d, J = 12.36 Hz, 3H). LC−MS: calculated for $C_{44}H_{53}N_4O_7$ $[M+H]^+$: 749.38, found 749.26.

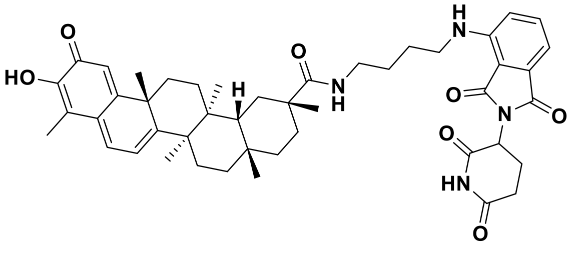

(2R,4aS,6aR,12bR,14aS,14bR)-N-(4-((2-(2,6-dioxopiperidin-3-yl)-1,3-dioxoisoindolin-4-yl)amino)butyl)-10-hydroxy-2,4a,6a,9,12b,14a-hexamethyl-11-oxo-1,2,3,4,4a,5,6,6a,11,12b,13,14,14a,14b-tetradecahydropicene-2-carboxamide (ZH-002). ^{1}H-NMR (400 MHz, $CDCl_3$) δ 8.26 (s, 1H), 7.45 (t, J = 7.8 Hz, 1H), 7.07 (d, J = 7 Hz, 1H), 6.99 (d, J = 6.96 Hz, 1H), 6.83 (d, J = 8.48 Hz, 1H), 6.52 (s, 1H), 6.33 (d, J = 7 Hz, 1H), 6.18 (s, 1H), 5.81 (s, 1H), 4.93-4.89 (m, 1H), 3.25-3.07 (m, 4H), 2.90-2.73 (m, 3H), 2.45 (d, J = 14.88 Hz, 1H), 2.19-1.25 (m, 28H), 1.13 (s, 3H), 1.11 (s, 3H), 1.02-0.99 (m, 1H), 0.62 (s, 3H). ^{13}C-NMR (400 MHz, $CDCl_3$) δ 178.34, 177.90, 170.95, 170.19, 169.52, 168.34, 167.55, 164.75, 146.82, 146.03, 136.24, 134.02, 132.44, 127,42, 119.54, 118.04, 117.09, 116.67, 111.63, 109.98, 48.88, 45.05, 44.33, 43.01, 42.14, 40.35, 39.34, 39.22, 38.19, 36.34, 35.05, 33.87, 33.44, 31.61, 31.43, 31.04, 30.86, 30.17, 29.71, 29.47, 26.83, 26.63, 22.82, 21.80, 18.39, 10.28. LC−MS: calculated for $C_{46}H_{57}N_4O_7$ $[M+H]^+$: 777.41, found 777.39.

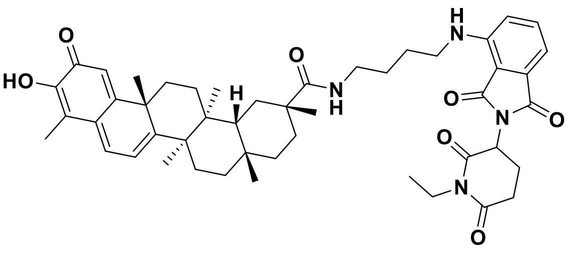

(2R,4aS,6aR,12bR,14aS,14bR)-N-(4-((2-(1-ethyl-2,6-dioxopiperidin-3-yl)-1,3-dioxoisoindolin-4-yl)amino)butyl)-10-hydroxy-2,4a,6a,9,12b,14a-hexamethyl-11-oxo-1,2,3,4,4a,5,6,6a,11,12b,13,14,14a,14b-tetradecahydropicene-2-carboxamide (ZH-002-N). ^{1}H-NMR (400 MHz, $CDCl_3$) δ 7.45 (t, *J* = 7.72 Hz, 1H), 7.06 (d, *J* = 7.04 Hz, 1H), 7.00 (d, *J* = 6.88 Hz, 1H), 6.96 (s, 1H), 6.83 (d, *J* = 8.48 Hz, 1H), 6.51 (s, 1H), 6.33 (d, *J* = 6.92 Hz, 1H), 6.17 (s, 1H), 5.80 (s, 1H), 4.90-4.85 (m, 1H), 3.25-3.15 (m, 6H), 2.95-2.70 (m, 3H), 2.45 (d, *J* = 15.08 Hz, 1H), 2.19 (s, 3H), 2.11-1.43 (m, 21H), 1.25 (s, 3H), 1.16-1.11 (m, 9H), 1.02-0.99 (m, 1H), 0.62 (s, 3H). LC−MS: calculated for $C_{48}H_{61}N_4O_7$ $[M+H]^+$: 805.45, found 805.71.

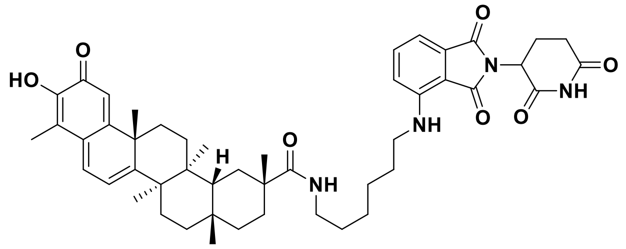

(2R,4aS,6aR,12bR,14aS,14bR)-N-(6-((2-(2,6-dioxopiperidin-3-yl)-1,3-dioxoisoindolin-4-yl)amino)hexyl)-10-hydroxy-2,4a,6a,9,12b,14a-hexamethyl-11-oxo-1,2,3,4,4a,5,6,6a,11,12b,13,14,14a,14b-tetradecahydropicene-2-carboxamide (ZH-003). ^{1}H-NMR (400 MHz, $CDCl_3$) δ 8.25 (d, *J* = 36.08 Hz, 1H), 7.48 (t, d = 7.4 Hz, 1H), 7.08 (d, *J* = 7.08 Hz, 1H), 7.00 (d, *J* = 7.04 Hz, 1H), 6.83 (d, *J* = 8.48 Hz, 1H), 6.52 (s, 1H), 6.33 (d, *J* = 7.16 Hz, 1H), 6.19 (s, 1H), 5.66 (s, 1H), 4.93-4.88 (m, 1H), 3.21 (q, *J* = 6.04 Hz, 2H), 3.17-3.02 (m, 2H), 2.91-2.68 (m, 3H), 2.43 (d, *J* = 15.16 Hz, 1H), 2.22 (s, 3H), 2.14-1.25 (m, 28H), 1.14 (s, 3H), 1.11 (s, 3H), 1.03-0.99 (s, 1H), 0.63 (s, 3H). LC−MS: calculated for $C_{48}H_{61}N_4O_7$ $[M+H]^+$: 805.45, found 805.43.

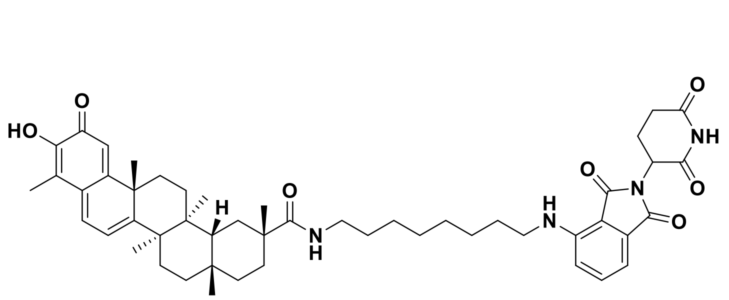

(2R,4aS,6aR,12bR,14aS,14bR)-N-(8-((2-(2,6-dioxopiperidin-3-yl)-1,3-dioxoisoindolin-4-yl)amino)octyl)-10-hydroxy-2,4a,6a,9,12b,14a-hexamethyl-11-oxo-1,2,3,4,4a,5,6,6a,11,12b,13,14,14a,14b-tetradecahydropicene-2-carboxamide (ZH-004). ^{1}H-

NMR (400 MHz, $CDCl_3$) δ 8.42-8.26 (m, 1H), 7.48 (t, d = 7.76 Hz, 1H), 7.08 (d, J = 7.12 Hz, 1H), 7.00 (d, J = 6.92 Hz, 1H), 6.86 (d, J = 8.52 Hz, 1H), 6.53 (s, 1H), 6.33 (d, J = 7.16 Hz, 1H), 6.21-6.20 (m, 1H), 5.64-5.63 (m, 1H), 4.93-4.89 (m, 1H), 3.22 (q, J = 6.04 Hz, 2H), 3.12-3.07 (m, 2H), 2.91-2.73 (m, 3H), 2.43 (d, J = 14.96 Hz, 1H), 2.19 (s, 3H), 2.14-1.25 (m, 32H), 1.13 (s, 3H), 1.11 (s, 3H), 1.02-0.99 (m, 1H), 0.63 (s, 3H). LC−MS: calculated for $C_{50}H_{65}N_4O_7$ $[M+H]^+$: 833.48, found 833.66.

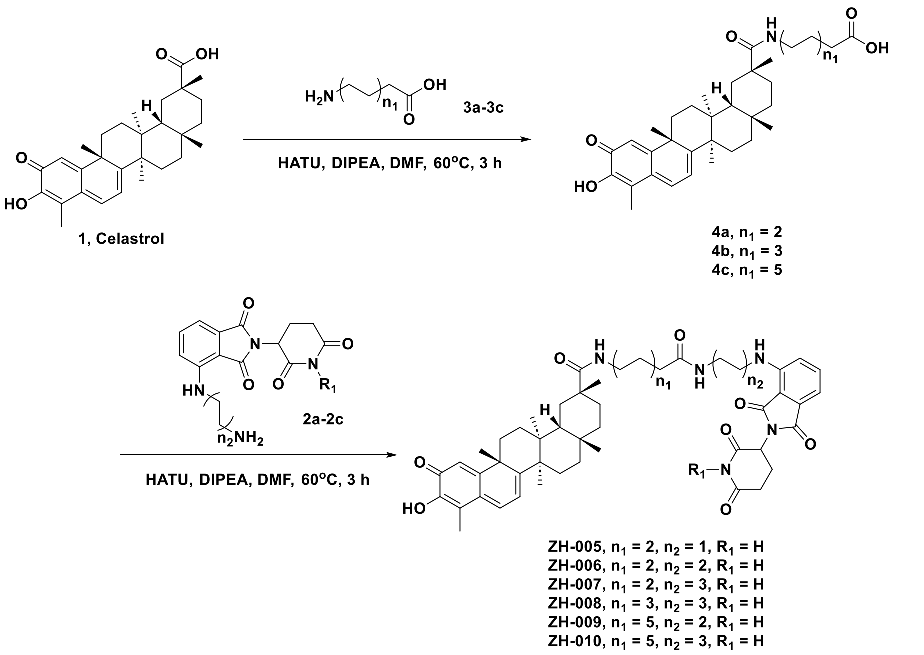

Celastrol (27 mg, 0.06 mmol), HATU (25.08 mg, 0.066 mmol, 1.1 eq.), DIPEA (30 μL, 0.18 mmol, 3 eq.), were dissolved in DMF and stirred at room temperature for 5 min. The compounds 3a-3c (0.054 mmol, 0.9 eq.) in DMF were added respectively, the mixture was stirred at 60°C for 3 h. Next, HATU (25.08 mg, 0.066 mmol, 1.1 eq.), DIPEA (10 μL, 0.06 mmol, 1eq.) were added and stirred for 5 min again, followed by the addition of compounds 2a-2c (0.06 mmol, 1eq.). The solution was stirred for another 3 h. The resulting mixture was poured into water and extracted with ethyl acetate, the organic layer was concentrated and further purified by silica gel column chromatography (DCM: MeOH = 50: 1 to DCM: MeOH = 30: 1) to give compounds ZH-005, ZH-006, ZH-007, ZH-008, ZH-009, ZH-010, IY = 40%.

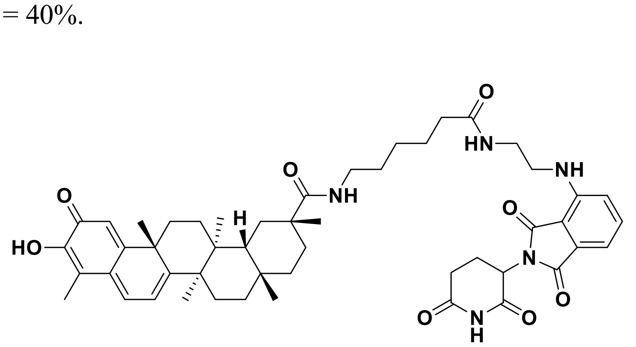

(2R,4aS,6aR,12bR,14aS,14bR)-N-(6-((2-((2-(2,6-dioxopiperidin-3-yl)-1,3-dioxoisoindolin-4-

yl)amino)ethyl)amino)-6-oxohexyl)-10-hydroxy-2,4a,6a,9,12b,14a-hexamethyl-11-oxo-1,2,3,4,4a,5,6,6a,11,12b,13,14,14a,14b-tetradecahydropicene-2-carboxamide (ZH-005). ^{1}H-NMR (400 MHz, $CDCl_3$) δ 8.33-8.27 (m, 1H), 7.51-7.46 (s, 1H), 7.18-7.15 (m, 1H), 7.10 (d, *J* = 7.64 Hz, 1H), 7.05 (d, *J* = 6.96 Hz, 1H), 6.97 (d, *J* = 8.52 Hz, 1H), 6.92 (s, 1H), 6.58 (s, 1H), 6.48 (s, 1H), 6.35 (d, *J* = 7.2 Hz, 1H), 5.60-5.59 (m, 1H), 4.95-4.91 (m, 1H), 3.56-3.00 (m, 6H), 2.90-2.74 (m, 3H), 2.26 (d, *J* = 15.52 Hz, 1H), 2.21 (s, 3H), 2.18-1.25 (m, 28H), 1.13 (s, 3H), 1.10 (s, 3H), 0.99-0.96 (m, 1H), 0.60 (s, 3H). LC−MS: calculated for $C_{50}H_{64}N_5O_8$ $[M+H]^+$: 862.47, found 862.74.

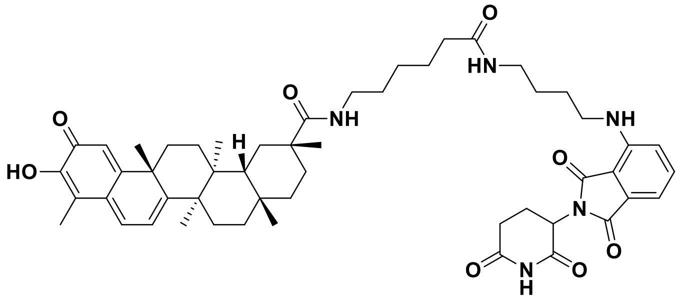

(2R,4aS,6aR,12bR,14aS,14bR)-N-(6-((4-((2-(2,6-dioxopiperidin-3-yl)-1,3-dioxoisoindolin-4-yl)amino)butyl)amino)-6-oxohexyl)-10-hydroxy-2,4a,6a,9,12b,14a-hexamethyl-11-oxo-1,2,3,4,4a,5,6,6a,11,12b,13,14,14a,14b-tetradecahydropicene-2-carboxamide (ZH-006). ^{1}H-NMR (400 MHz, $CDCl_3$) δ 8.30-8.24 (m, 1H), 7.48 (t, *J* = 7.6 Hz, 1H), 7.08 (d, *J* = 7.04 Hz, 1H), 7.03-6.99 (m, 2H), 6.88 (d, *J* = 8.52 Hz, 1H), 6.51 (s, 1H), 6.34 (d, *J* = 7.16 Hz, 1H), 6.24 (t, *J* = 5.04 Hz, 1H), 6.06 (s, 1H), 5.67-5.66 (m, 1H), 4.94-4.90 (m, 1H), 3.30-3.01 (m, 6H), 2.91-2.69 (m, 3H), 2.35 (d, *J* = 15.88 Hz, 1H), 2.20 (s, 3H), 2.15-1.25 (m, 32H), 1.13 (s, 3H), 1.11 (s, 3H), 1.00-0.97 (m, 1H), 0.62 (s, 3H). LC−MS: calculated for $C_{52}H_{68}N_5O_8$ $[M+H]^+$: 890.50, found 890.78.

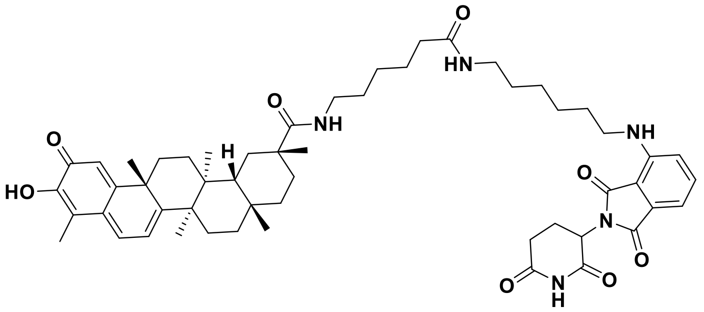

(2R,4aS,6aR,12bR,14aS,14bR)-N-(6-((6-((2-(2,6-dioxopiperidin-3-yl)-1,3-dioxoisoindolin-4-yl)amino)hexyl)amino)-6-oxohexyl)-10-hydroxy-2,4a,6a,9,12b,14a-hexamethyl-11-oxo-1,2,3,4,4a,5,6,6a,11,12b,13,14,14a,14b-tetradecahydropicene-2-carboxamide (ZH-007). ^{1}H-NMR (400 MHz, $CDCl_3$) δ 8.23 (s, 1H), 7.49 (t, *J* = 7.56 Hz, 1H), 7.08 (d, *J* = 7 Hz, 1H), 7.03-6.99 (m, 2H), 6.87 (d, *J* = 8.56 Hz, 1H), 6.52 (s, 1H), 6.34 (d, *J* = 7.12 Hz, 1H), 6.22 (t, *J* = 5.4 Hz, 1H), 5.85 (s, 1H), 5.70 (t, *J* = 4.8 Hz, 1H), 4.93-4.89 (m, 1H), 3.28-3.03 (m, 6H), 2.91-2.69 (m, 3H), 2.38 (d, *J* = 15.48 Hz, 1H), 2.21 (s, 3H), 2.15-1.25 (m, 36H), 1.14 (s, 3H), 1.11 (s, 3H), 1.02-0.98 (m, 1H), 0.62 (s, 3H). LC−MS: calculated for $C_{54}H_{72}N_5O_8$ $[M+H]^+$: 918.53, found 918.82.

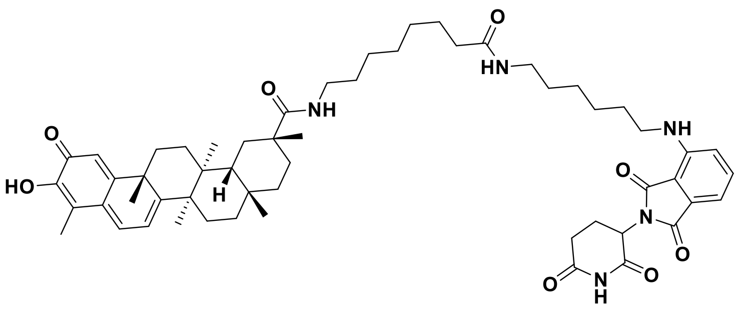

(2R,4aS,6aR,12bR,14aS,14bR)-N-(8-((6-((2-(2,6-dioxopiperidin-3-yl)-1,3-dioxoisoindolin-4-yl)amino)hexyl)amino)-8-oxooctyl)-10-hydroxy-2,4a,6a,9,12b,14a-hexamethyl-11-oxo-1,2,3,4,4a,5,6,6a,11,12b,13,14,14a,14b-tetradecahydropicene-2-carboxamide (ZH-008). H-NMR (400 MHz, $CDCl_3$) δ 8.24-8.20 (m, 1H), 7.48 (t, *J* = 7.48 Hz, 1H), 7.08 (d, *J* = 7.08 Hz, 1H), 7.03 (d, *J* = 7 Hz, 1H), 6.99 (s, 1H), 6.87 (d, *J* = 8.56 Hz, 1H), 6.51 (s, 1H), 6.35 (d, *J* = 7.16 Hz, 1H), 6.22 (t, *J* = 5.48 Hz, 1H), 5.99 (s, 1H), 5.52 (t, *J* = 5.32 Hz, 1H), 4.93-4.89 (m, 1H), 3.28-2.98 (m, 6H), 2.92-2.68 (m, 3H), 2.36 (d, *J* = 14.96 Hz, 1H), 2.20 (s, 3H), 2.15-1.25 (m, 40H), 1.14 (s, 3H), 1.11 (s, 3H), 1.02-0.98 (m, 1H), 0.63 (s, 3H). LC−MS: calculated for $C_{56}H_{76}N_5O_8$ $[M+H]^+$: 946.56, found 946.95.

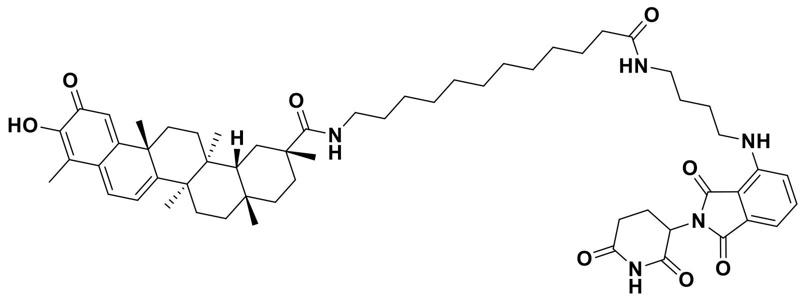

(2R,4aS,6aR,12bR,14aS,14bR)-N-(12-((4-((2-(2,6-dioxopiperidin-3-yl)-1,3-dioxoisoindolin-4-yl)amino)butyl)amino)-12-oxododecyl)-10-hydroxy-2,4a,6a,9,12b,14a-hexamethyl-11-oxo-1,2,3,4,4a,5,6,6a,11,12b,13,14,14a,14b-tetradecahydropicene-2-carboxamide (ZH-009). ^{1}H-NMR (400 MHz, $CDCl_3$) δ 8.28-8.24 (m, 1H), 7.49 (t, *J* = 7.88 Hz, 1H), 7.09 (d, *J* = 7.12 Hz, 1H), 7.01-6.99 (m, 2H), 6.89 (d, *J* = 8.48 Hz, 1H), 6.52 (s, 1H), 6.33 (d, *J* = 7.16 Hz, 1H), 6.23 (t, *J* = 5.44 Hz, 1H), 5.66 (m, 2H), 4.94-4.89 (m, 1H), 3.22-3.05 (m, 6H), 2.91-2.72 (m, 3H), 2.44 (d, *J* = 14.96 Hz, 1H), 2.20 (s, 3H), 2.16-1.19 (m, 44H), 1.14 (s, 3H), 1.11 (s, 3H), 1.03-0.99 (m, 1H), 0.63 (s, 3H). LC−MS: calculated for $C_{58}H_{80}N_5O_8$ $[M+H]^+$: 974.59, found 974.99.

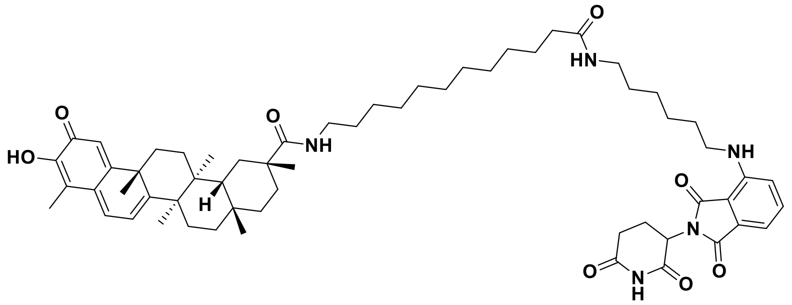

(2R,4aS,6aR,12bR,14aS,14bR)-N-(12-((6-((2-(2,6-dioxopiperidin-3-yl)-1,3-dioxoisoindolin-4-yl)amino)hexyl)amino)-12-oxododecyl)-10-hydroxy-2,4a,6a,9,12b,14a-hexamethyl-11-oxo-1,2,3,4,4a,5,6,6a,11,12b,13,14,14a,14b-tetradecahydropicene-2-carboxamide (ZH-010). ^{1}H-NMR (400 MHz, $CDCl_3$) δ 8.22-8.20 (m, 1H), 7.49 (t, J = 7.64 Hz, 1H), 7.08 (d, J = 7.04 Hz, 1H), 7.01-7.00 (m, 2H), 6.87 (d, J = 8.52 Hz, 1H), 6.52 (s, 1H), 6.33 (d, J = 7.16 Hz, 1H), 6.22 (t, J = 5.32 Hz, 1H), 5.66 (t, J = 5.24 Hz, 1H), 5.57 (s, 1H), 4.93-4.89 (m, 1H), 3.28-3.00 (m, 6H), 2.91-2.69 (m, 3H), 2.44 (d, J = 14.76 Hz, 1H), 2.20 (s, 3H), 2.15-1.20 (m, 44H), 1.14 (s, 3H), 1.12 (s, 3H), 1.03-0.99 (m, 1H), 0.63 (s, 3H). LC−MS: calculated for $C_{60}H_{84}N_5O_8$ $[M+H]^+$: 1002.62, found 1002.93.

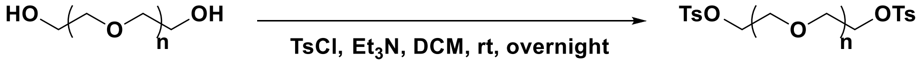

5a-5e 6a-6e

Compounds 5a-5e (10 mmol) and tosyl chloride (4.2 g, 22 mmol, 2.2 eq.) were dissolved in DCM. Then Et_3N (4.2 mL, 30 mmol, 3 eq.) was added dropwise at 0°C. The mixture was warmed up to room temperature and stirred overnight. The resulting mixture was poured into water and extracted with DCM, the organic layer was concentrated and recrystallized using petroleum ether-ethyl acetate to give products 6a-6e, IY = 80%.

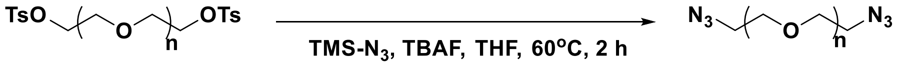

6a-6e 7a-7e

Trimethylsilylmethyl azide (2.6 mL, 17.6 mmol, 2.2 eq.), TBAF (17.6 mL, 17.6 mmol, 2.2 eq.) were added to a solution of compounds 6a-6e (8 mmol) in THF. The reaction was stirred at 60°C for 2 h. The resulting mixture was concentrated in vacuo and further purified by silica gel column chromatography (PE: EA = 5: 1) to give compounds 7a-7e, IY = 60%.

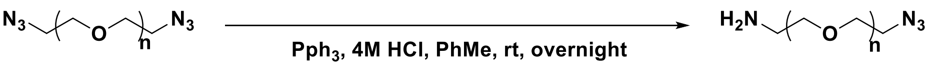

7a-7e 8a-8e

Compounds 7a-7e (4 mmol) were dissolved in a mixed HCl/PhMe (1:1, v/v) solution. Pph3 (942

mg, 3.6 mmol, 0.9 eq.) was added portionwise at 0°C and the solution was warmed up to room temperature and stirred overnight. The resulting mixture was washed by DCM for 3 times and the aqueous phase was basified with 2M NaOH solution. Then the aqueous phase was extracted with ethyl acetate for 4 times, the organic layer was concentrated to give crude compounds 8a-8e without further purification, IY = 50%.

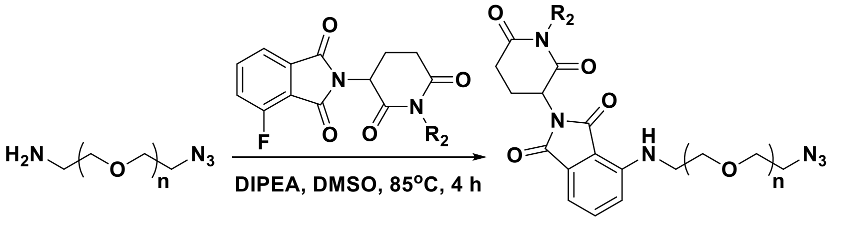

8a-8e

9a, n = 1, R_2 = H; 9a', n = 1, R_2 = Et
9b, n = 2, R_2 = H
9c, n = 3, R_2 = H; 9c', n = 3, R_2 = Et
9d, n = 4, R_2 = H
9e, n = 5, R_2 = H; 9e', n = 5, R_2 = Et

Pomalidomide analogues (276 mg, 1 mmol), compounds 8a-8e (2 mmol, 2 eq.), DIPEA (330 μL, 2 mmol, 2 eq.) were dissolved in DMSO. The mixture was stirred at 85°C for 4 h. The solution was washed with water and extracted with ethyl acetate, the organic layer was concentrated and further purified by silica gel column chromatography (DCM: MeOH = 50: 1 to DCM: MeOH = 30: 1) to give compounds 9a-9e, 9a’, 9c’, 9e’, IY = 25%.

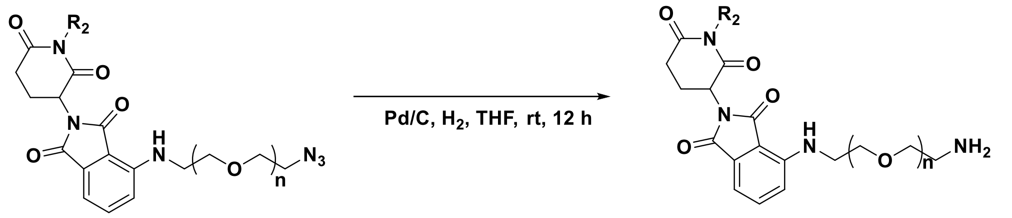

9a, n = 1, R_2 = H; 9a', n = 1, R_2 = Et
9b, n = 2, R_2 = H
9c, n = 3, R_2 = H; 9c', n = 3, R_2 = Et
9d, n = 4, R_2 = H
9e, n = 5, R_2 = H; 9e', n = 5, R_2 = Et

10a, n = 1, R_2 = H; 10a', n = 1, R_2 = Et
10b, n = 2, R_2 = H
10c, n = 3, R_2 = H; 10c', n = 3, R_2 = Et
10d, n = 4, R_2 = H
10e, n = 5, R_2 = H; 10e', n = 5, R_2 = Et

The solution of compounds 9a-9e, 9a’, 9c’, 9e’ (0.1 mmol) and 10% Pd/C in THF was stirred at room temperature under a hydrogen atmosphere for 12 h. The resulting mixture was filtered through a Celite pad and concentrated to give crude intermediates 10a-10e, 10a’, 10c’, 10e’ without further purification, IY = 60%.

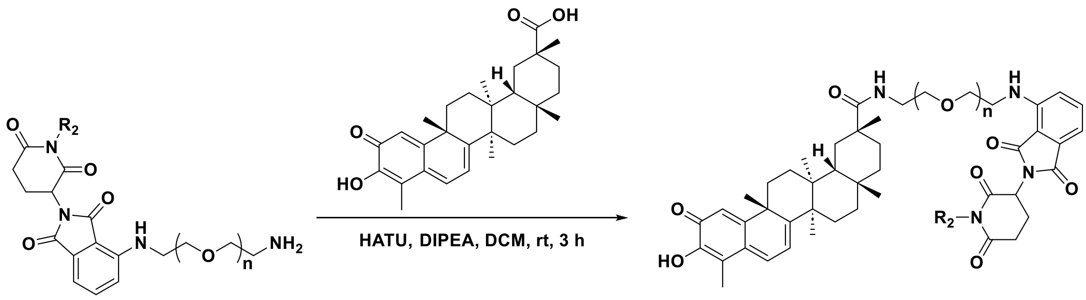

Celastrol (13.5 mg, 0.03 mmol), HATU (12.54 mg, 0.033 mmol, 1.1 eq.), DIPEA (15 μL, 0.09 mmol, 3 eq.) were dissolved in DCM and stirred at room temperature for 5 min. Then the compounds 10a-10e, 10a', 10c', 10e' (0.03 mmol, 1 eq.) in DCM were added respectively. The mixture was stirred for another 3 h. The resulting mixture was poured into water and extracted with DCM, the organic layer was concentrated and further purified by silica gel column chromatography (DCM: MeOH = 50: 1 to DCM: MeOH = 30: 1) to give compounds ZH-011, ZH-011-N, ZH-012, ZH-013, ZH-013-N, ZH-014, ZH-015, ZH-015-N, IY = 45%-55%.

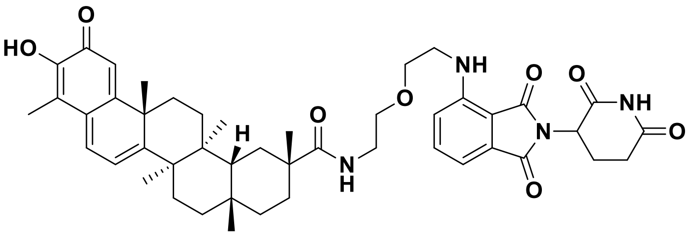

(2R,4aS,6aR,12bR,14aS,14bR)-N-(2-(2-((2-(2,6-dioxopiperidin-3-yl)-1,3-dioxoisoindolin-4-yl)amino)ethoxy)ethyl)-10-hydroxy-2,4a,6a,9,12b,14a-hexamethyl-11-oxo-1,2,3,4,4a,5,6,6a,11,12b,13,14,14a,14b-tetradecahydropicene-2-carboxamide (ZH-011). ^{1}H-NMR (400 MHz, $CDCl_3$,) δ 8.21 (s, 1H), 7.53 (t, J = 7.88 Hz, 1H), 7.14 (d, J = 7.08 Hz, 1H), 7.01-6.94 (m, 3H), 6.54 (t, J = 4.92 Hz, 1H), 6.49 (s, 1H), 6.31 (dd, J_1 = 7.12 Hz, J_2 = 2.4 Hz, 1H), 6.16 (q, J = 5.04 Hz, 1H), 4.95-4.91 (m, 1H), 3.70-3.11 (m, 8H), 2.93-2.75 (m, 3H), 2.35 (d, J = 13.56 Hz, 1H), 2.20 (s, 3H), 2.15-1.22 (m, 20H), 1.11 (s, 3H), 1.09 (s, 3H), 0.95-0.92 (m, 1H), 0.61 (s, 3H). ^{13}C-NMR (400 MHz, $CDCl_3$) δ 178.25, 177.93, 171.06, 170.46, 169.43, 168.31, 167.47, 164.82, 146.81, 145.98, 136.19, 134.25, 132.42, 127,27, 119.39, 118.00, 117.23, 116.77, 111.82, 110.29, 69.65, 69.27, 48.89, 45.01, 44.32, 43.37, 42.96, 42.22, 40.14, 39.20, 38.10, 36.27, 34.86, 33.67, 33.49, 31.55, 31.42, 30.14, 30.66, 29.94, 28.60, 22.72, 21.64, 18.29, 12.49, 10.22. LC−MS: calculated for $C_{46}H_{57}N_4O_8$ $[M+H]^+$: 793.41, found 793.45.

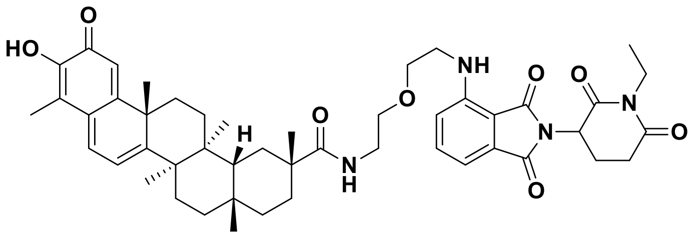

(2R,4aS,6aR,12bR,14aS,14bR)-N-(2-(2-((2-(1-ethyl-2,6-dioxopiperidin-3-yl)-1,3-dioxoisoindolin-4-yl)amino)ethoxy)ethyl)-10-hydroxy-2,4a,6a,9,12b,14a-hexamethyl-11-oxo-1,2,3,4,4a,5,6,6a,11,12b,13,14,14a,14b-tetradecahydropicene-2-carboxamide (ZH-011-N). ^{1}H-NMR (400 MHz, $CDCl_3$) δ 7.52 (t, J = 7.92 Hz, 1H), 7.13 (d, J = 7.08 Hz, 1H), 7.00-6.94 (m, 3H), 6.54 (s, 1H), 6.48 (d, J = 3.48 Hz, 1H), 6.30 (dd, J_1 = 7.16 Hz, J_2 = 1.96 Hz, 1H), 6.18-6.13 (m, 1H), 4.91-4.87 (m, 1H), 3.86 (q, J = 7.12 Hz, 2H), 3.64 (t, J = 5 Hz, 2H), 3.50-3.33 (m, 6H), 2.98-2.73 (m, 3H), 2.34 (d, J = 15.04 Hz, 1H), 2.20 (s, 3H), 2.10-1.22 (m, 20H), 1.15 (t, J = 7.04 Hz, 3H), 1.10 (d, J = 1.72 Hz, 3H), 1.08 (s, 3H), 0.95-0.91 (m, 1H), 0.61 (s, 3H). LC−MS: calculated for $C_{48}H_{61}N_4O_8$ $[M+H]^+$: 821.44, found 821.87.

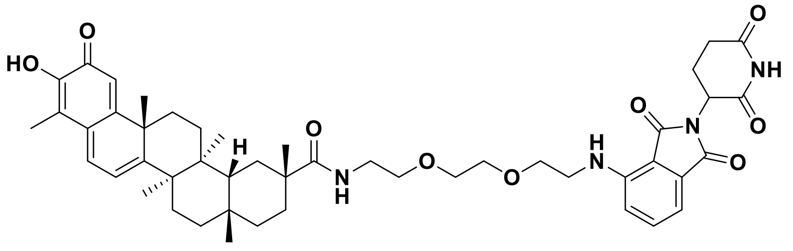

(2R,4aS,6aR,12bR,14aS,14bR)-N-(2-(2-(2-((2-(2,6-dioxopiperidin-3-yl)-1,3-dioxoisoindolin-4-yl)amino)ethoxy)ethoxy)ethyl)-10-hydroxy-2,4a,6a,9,12b,14a-hexamethyl-11-oxo-1,2,3,4,4a,5,6,6a,11,12b,13,14,14a,14b-tetradecahydropicene-2-carboxamide (ZH-012). ^{1}H-NMR (400 MHz, $CDCl_3$) δ 8.47-8.36 (m, 1H), 7.49 (t, J = 7.84 Hz, 1H), 7.11 (d, J = 7.12 Hz, 1H), 7.00-6.96 (m, 2H), 6.89 (d, J = 8.4 Hz, 1H), 6.51 (s, 1H), 6.48 (t, J = 5 Hz, 1H), 6.31 (d, J = 7.16 Hz, 1H), 6.15 (q, J = 6.56 Hz, 1H), 4.90-4.86 (m, 1H), 3.70 (t, J = 5.16 Hz, 2H), 3.63-3.62 (m, 4H), 3.51 (t, J = 4.92 Hz, 2H), 3.45 (q, J = 5.32 Hz, 2H), 3.33-3.32 (m, 2H), 2.88-2.71 (m, 3H), 2.44 - 2.38(m, 1H), 2.19 (d, J = 3.64 Hz, 3H), 2.14-1.24 (m, 20H), 1.13 (s, 3H), 1.10 (s, 3H), 0.99-0.95 (m, 1H), 0.63 (s, 3H). LC−MS: calculated for $C_{48}H_{61}N_4O_9$ $[M+H]^+$: 837.44, found 837.36.

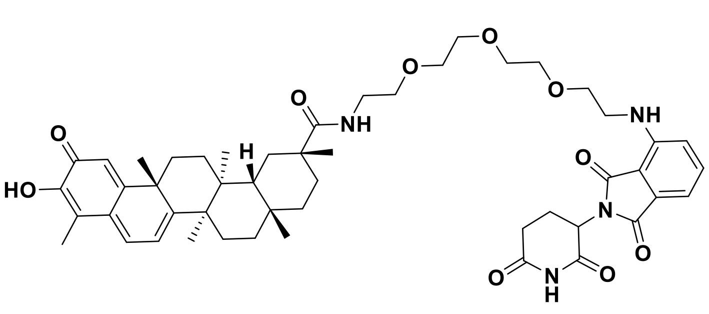

(2R,4aS,6aR,12bR,14aS,14bR)-N-(2-(2-(2-(2-((2-(2,6-dioxopiperidin-3-yl)-1,3-dioxoisoindolin-4-yl)amino)ethoxy)ethoxy)ethoxy)ethyl)-10-hydroxy-2,4a,6a,9,12b,14a-

hexamethyl-11-oxo-1,2,3,4,4a,5,6,6a,11,12b,13,14,14a,14b-tetradecahydropicene-2-carboxamide (ZH-013). ^{1}H-NMR (400 MHz, $CDCl_3$) δ 8.49 (d, *J* = 24.68 Hz, 1H), 7.48 (t, *J* = 8.04 Hz, 1H), 7.10 (d, *J* = 6.96 Hz, 1H), 7.00 (d, *J* = 6.8 Hz, 1H), 6.91 (d, *J* = 8.44 Hz, 1H), 6.51 (s, 1H), 6.49 (s, 1H), 6.32 (d, *J* = 6.92 Hz, 1H), 6.24 (s, 1H), 4.92-4.89 (m, 1H), 3.71-3.45 (m, 14H), 3.31 (d, *J* = 3.96 Hz, 2H), 2.89-2.73 (m, 3H), 2.42 (d, *J* = 15.52 Hz, 1H), 2.19 (s, 3H), 2.13-1.25 (m, 20H) , 1.13 (s, 3H), 1.10 (s, 3H), 1.00-0.96 (m, 1H), 0.62 (s, 3H). ^{13}C-NMR (400 MHz, $CDCl_3$) δ 178.31, 177.81, 171.03, 170.29, 169.25, 168.33, 167.56, 164.74, 146.78, 146.00, 136.04, 134.02, 132.52, 129.87, 127.37, 119.51, 117.99, 116.72, 111.70, 110.34, 70.76, 70.58, 70.52, 70.08, 69.74, 48.86, 45.03, 44.37, 42.98, 42.38, 40.23, 39.32, 39.17, 38.16, 36.36, 34.94, 33.74, 33.48, 31.60, 31.40, 31.14, 30.78, 30.03, 28.65, 22.80, 21.72, 18.30, 14.10, 10.24. LC−MS: calculated for $C_{50}H_{65}N_4O_{10}$ [M+H]$^+$: 881.46, found 881.94.

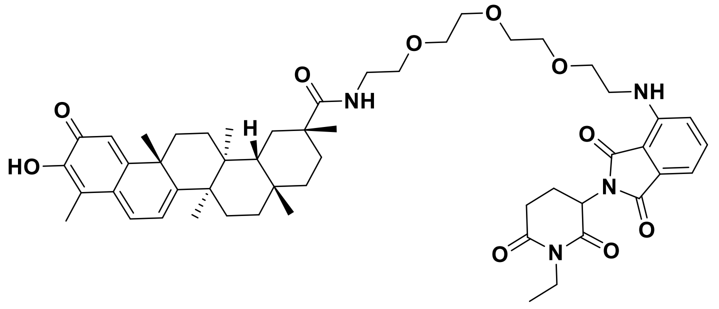

(2R,4aS,6aR,12bR,14aS,14bR)-N-(2-(2-(2-(2-((2-(1-ethyl-2,6-dioxopiperidin-3-yl)-1,3-dioxoisoindolin-4-yl)amino)ethoxy)ethoxy)ethoxy)ethyl)-10-hydroxy-2,4a,6a,9,12b,14a-hexamethyl-11-oxo-1,2,3,4,4a,5,6,6a,11,12b,13,14,14a,14b-tetradecahydropicene-2-carboxamide (ZH-013-N). ^{1}H-NMR (400 MHz, $CDCl_3$) δ 7.48 (t, *J* = 8 Hz, 1H), 7.10 (d, *J* = 7.12 Hz, 1H), 6.99 (d, *J* = 7.04 Hz, 1H), 6.91 (d, *J* = 8.56 Hz, 1H), 6.51 (s, 1H), 6.47 (t, *J* = 5.52 Hz, 1H), 6.32 (d, *J* = 7.16 Hz, 1H), 6.20 (t, *J* = 5 Hz, 1H), 4.91-4.86 (m, 1H), 3.85 (q, *J* = 7.04 Hz, 2H), 3.70 (t, *J* = 5.4 Hz, 2H), 3.65-3.46 (m, 12H), 3.31 (q, *J* = 4.96 Hz, 2H), 2.95-2.71 (m, 3H), 2.43 (d, *J* = 15.4 Hz, 1H), 2.19 (s, 3H), 2.14-1.24 (m, 20H), 1.14 (t, *J* = 6.96 Hz, 3H), 1.13 (s, 3H), 1.10 (s, 3H), 1.00-0.97 (m, 1H), 0.62 (s, 3H). LC−MS: calculated for $C_{52}H_{69}N_4O_{10}$ [M+H]$^+$: 909.49, found 909.88.

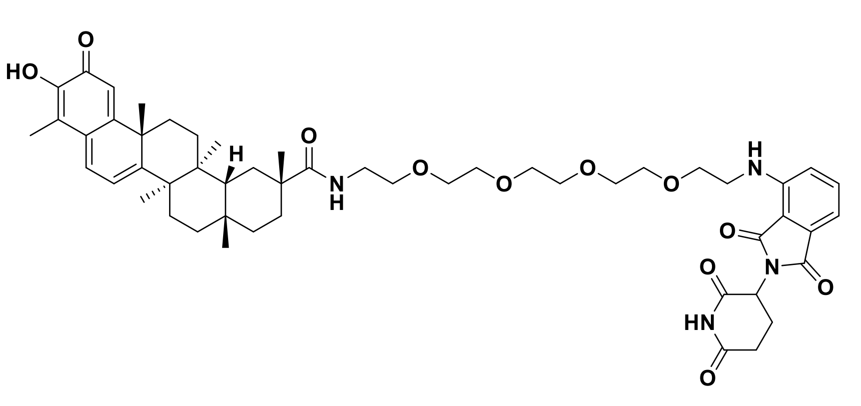

(2R,4aS,6aR,12bR,14aS,14bR)-N-(14-((2-(2,6-dioxopiperidin-3-yl)-1,3-dioxoisoindolin-4-yl)amino)-3,6,9,12-tetraoxatetradecyl)-10-hydroxy-2,4a,6a,9,12b,14a-hexamethyl-11-oxo-1,2,3,4,4a,5,6,6a,11,12b,13,14,14a,14b-tetradecahydropicene-2-carboxamide (ZH-014). ^{1}H-

NMR (400 MHz, $CDCl_3$) δ 8.53 (s, 1H), 7.48 (t, *J* = 7.44 Hz, 1H), 7.09 (d, *J* = 7.04 Hz, 1H), 7.02-6.98 (m, 2H), 6.91 (d, *J* = 8.52 Hz, 1H), 6.50 (s, 2H), 6.37 (d, *J* = 5.84 Hz, 1H), 6.32 (d, *J* = 7.04 Hz, 1H), 4.93-4.89 (m, 1H), 3.74-3.16 (m, 20H), 2.90-2.72 (m, 3H), 2.43-2.37 (m, 1H), 2.20 (s, 3H), 2.13-1.23 (m, 20H), 1.12 (s, 3H), 1.08 (d, *J* = 2.68 Hz, 3H), 0.99-0.95 (m, 1H), 0.60 (s, 3H). LC−MS: calculated for $C_{52}H_{69}N_4O_{11}$ $[M+H]^+$: 925.49, found 925.76.

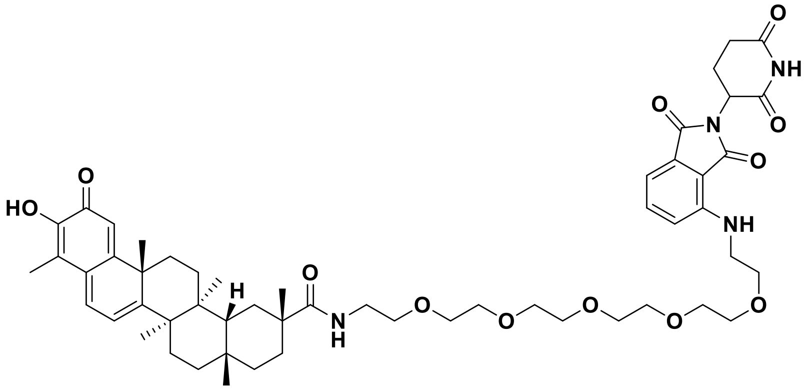

(2R,4aS,6aR,12bR,14aS,14bR)-N-(17-((2-(2,6-dioxopiperidin-3-yl)-1,3-dioxoisoindolin-4-yl)amino)-3,6,9,12,15-pentaoxaheptadecyl)-10-hydroxy-2,4a,6a,9,12b,14a-hexamethyl-11-oxo-1,2,3,4,4a,5,6,6a,11,12b,13,14,14a,14b-tetradecahydropicene-2-carboxamide (ZH-015). ^{1}H-NMR (400 MHz, $CDCl_3$) δ 8.61-8.54 (m, 1H), 7.49 (t, *J* = 7.92 Hz, 1H), 7.11 (d, *J* = 6.88 Hz, 1H), 7.00 (d, *J* = 5.8 Hz, 2H), 6.91 (d, *J* = 8.52 Hz, 1H), 6.52 (s, 2H), 6.38-6.32 (m, 2H), 4.94-4.89 (m, 1H), 3.73-3.28 (m, 24H), 2.90-2.73 (m, 3H), 2.45-2.39 (m, 1H), 2.20 (s, 3H), 2.14-1.22 (m, 20H), 1.13 (s, 3H), 1.09 (d, *J* = 4.76 Hz, 3H), 0.98-0.96 (m, 1H), 0.62 (d, *J* = 4.6 Hz, 3H). ^{13}C-NMR (400 MHz, $CDCl_3$) δ 178.31, 177.83, 171.17, 170.30, 169.22, 168.52, 167.57, 164.74, 146.79, 145.99, 136.01, 134.01, 132.55, 127,35, 119.52, 117.97, 117.05, 116.72, 111.62, 110.35, 70.79, 70.63, 70.58, 70.51, 70.30, 70.02, 69.91, 69.55, 69.46, 69.35, 48.87, 45.02, 44.38, 42.97, 42.35, 40.21, 39.31, 39.10, 38.15, 36.36, 34.95, 33.73, 33.50, 31.60, 31.45, 31.07, 30.74, 30.13, 28.64, 22.81, 21.71, 18.23, 14.10, 10.23. LC−MS: calculated for $C_{54}H_{73}N_4O_{12}$ $[M+H]^+$: 969.51, found 969.95.

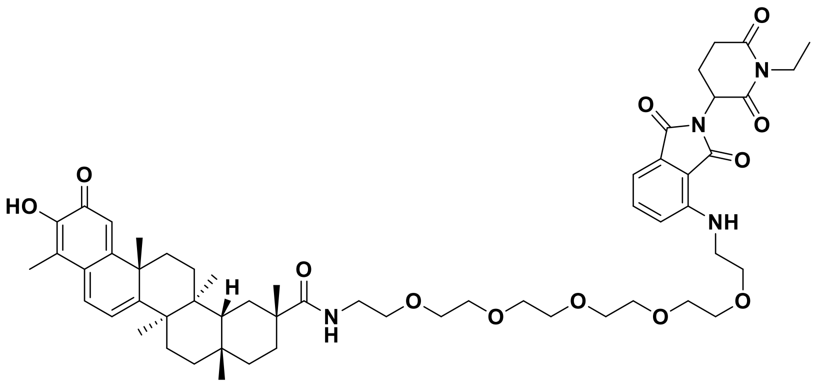

(2R,4aS,6aR,12bR,14aS,14bR)-N-(17-((2-(1-ethyl-2,6-dioxopiperidin-3-yl)-1,3-dioxoisoindolin-4-yl)amino)-3,6,9,12,15-pentaoxaheptadecyl)-10-hydroxy-2,4a,6a,9,12b,14a-hexamethyl-11-oxo-1,2,3,4,4a,5,6,6a,11,12b,13,14,14a,14b-tetradecahydropicene-2-

carboxamide (ZH-015-N). ^{1}H-NMR (400 MHz, $CDCl_3$) δ 7.48 (t, *J* = 7.32 Hz, 1H), 7.09 (d, *J* = 6.92 Hz, 1H), 7.00 (d, *J* = 7.12 Hz, 1H), 6.97 (s, 1H), 6.92 (d, *J* = 8.52 Hz, 1H), 6.50 (s, 1H), 6.46 (t, *J* = 5.32 Hz, 1H), 6.33 (d, *J* = 7.16 Hz, 1H), 6.24 (s, 1H), 4.90-4.86 (m, 1H), 3.89-3.83 (m, 2H), 3.73-3.10 (m, 24H), 2.96-2.71 (m, 3H), 2.44 (d, *J* = 15.12 Hz, 1H), 2.20 (s, 3H), 2.14-1.25 (m, 20H), 1.16-1.10 (m, 9H), 1.01-0.98 (m, 1H), 0.62 (s, 3H). LC−MS: calculated for $C_{56}H_{77}N_4O_{12}$ $[M+H]^+$: 997.55, found 997.71.

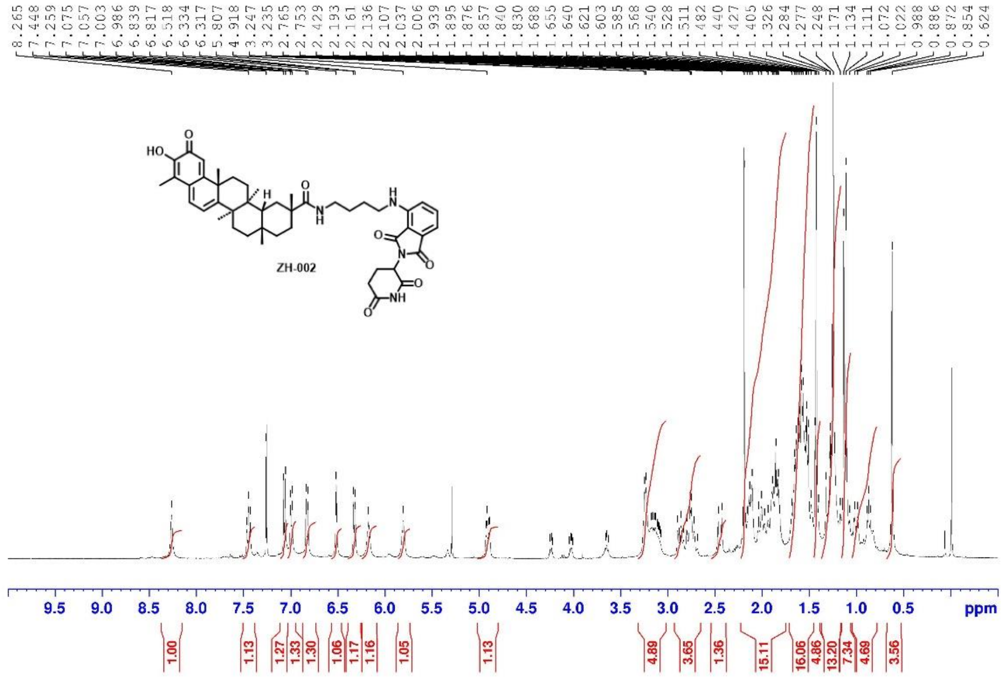

8.265
7.448
7.259
7.075
7.057
7.003
6.986
6.839
6.817
6.518
6.334
6.317
5.807
4.918
3.247
3.235
2.765
2.753
2.429
2.193
2.161
2.136
2.107
2.037
2.006
1.939
1.895
1.876
1.857
1.840
1.830
1.688
1.655
1.640
1.621
1.603
1.585
1.568
1.540
1.528
1.511
1.482
1.440
1.427
1.405
1.326
1.284
1.277
1.248
1.171
1.134
1.111
1.072
1.022
0.988
0.886
0.872
0.854
0.624
HO
O
H
N
NH
ZH-002
9.5 9.0 8.5 8.0 7.5 7.0 6.5 6.0 5.5 5.0 4.5 4.0 3.5 3.0 2.5 2.0 1.5 1.0 0.5 ppm
1.00
1.13
1.27
1.33
1.30
1.06
1.17
1.16
1.05
1.13
4.89
3.65
1.36
15.11
16.06
4.86
13.20
7.34
4.69
3.56

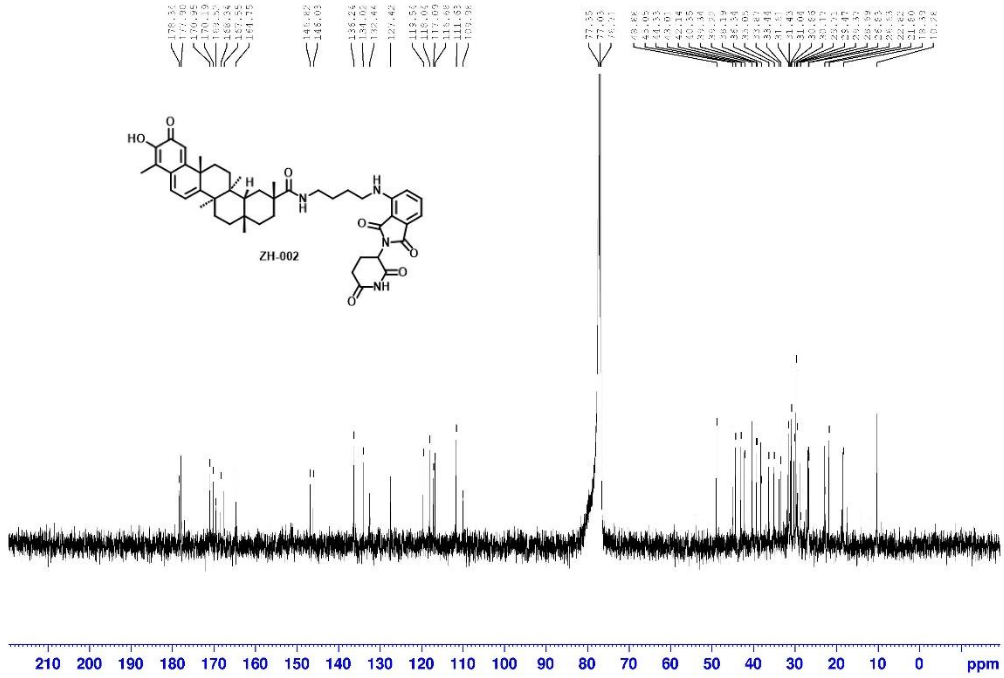

HO
O
H
N
NH
ZH-002
210 200 190 180 170 160 150 140 130 120 110 100 90 80 70 60 50 40 30 20 10 0 ppm

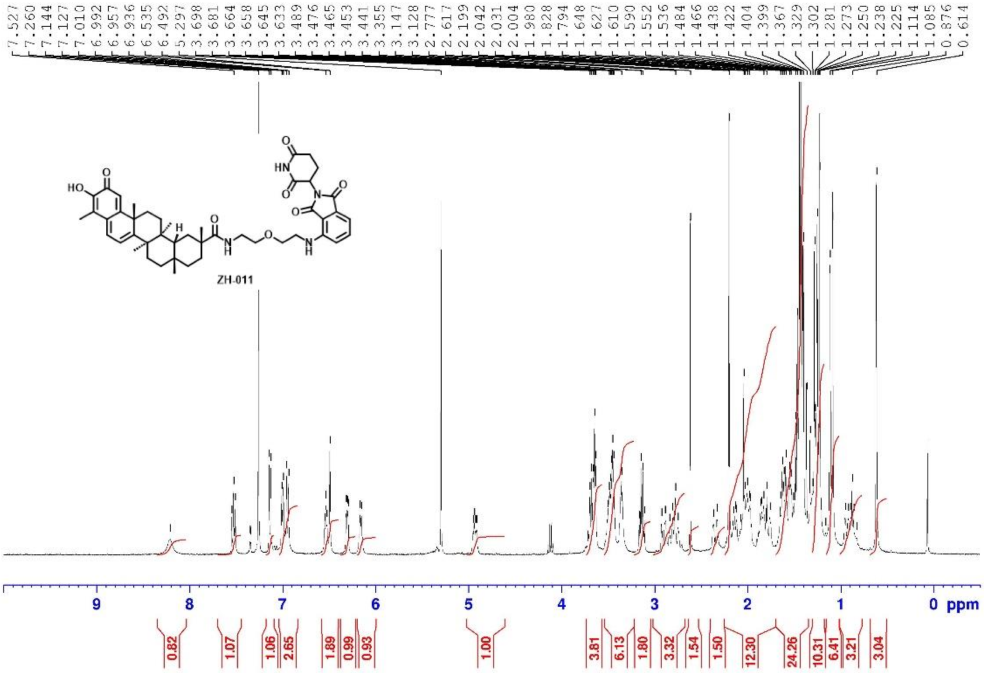
ZH-011
9 8 7 6 5 4 3 2 1 0 ppm
0.82 1.07 1.06 2.65 1.89 0.99 0.93 1.00 3.81 6.13 1.80 3.32 1.54 1.50 12.30 24.26 10.31 6.41 3.21 3.04

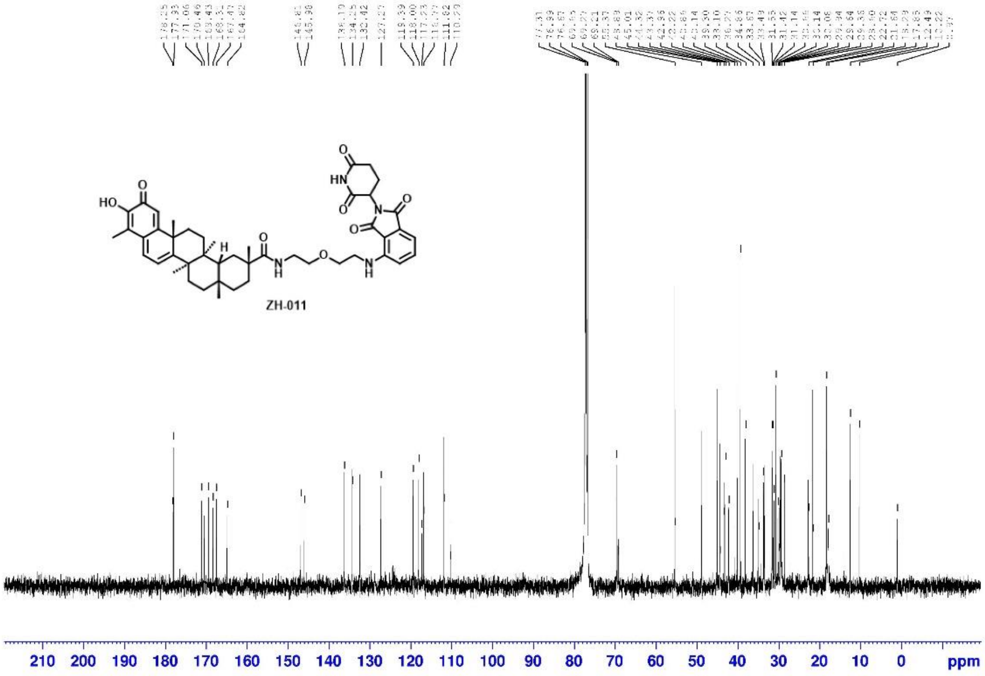
ZH-011
210 200 190 180 170 160 150 140 130 120 110 100 90 80 70 60 50 40 30 20 10 0 ppm

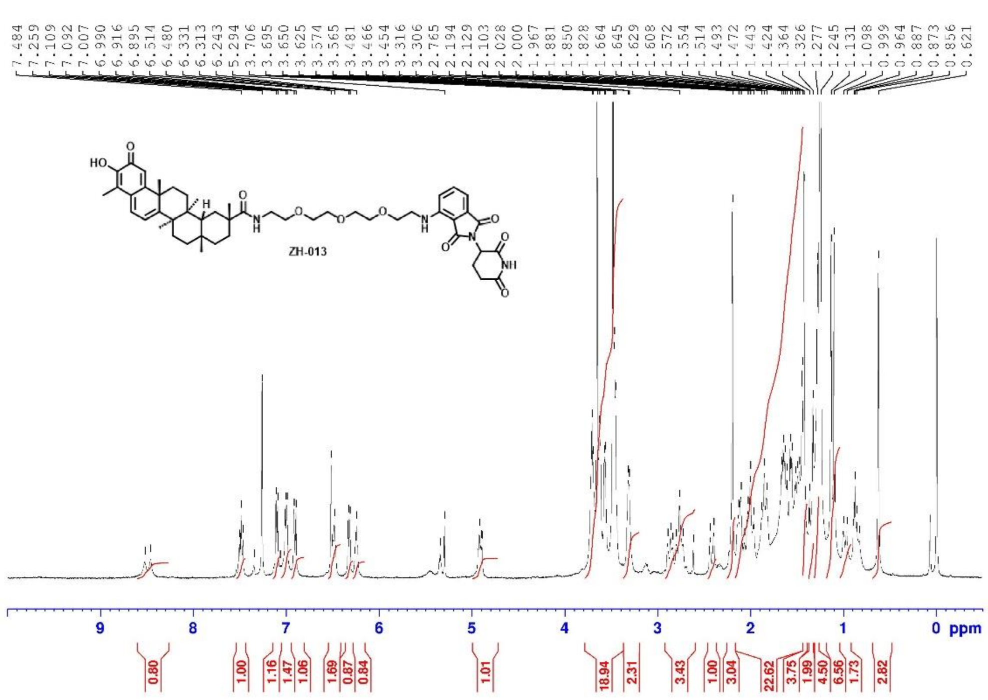
7.484 7.259 7.109 7.092 7.007 6.990 6.916 6.895 6.514 6.480 6.331 6.313 6.243 5.294 3.706 3.695 3.650 3.625 3.574 3.565 3.481 3.466 3.454 3.316 3.306 2.765 2.194 2.129 2.103 2.028 2.000 1.967 1.881 1.850 1.828 1.664 1.645 1.629 1.608 1.572 1.554 1.514 1.493 1.472 1.443 1.424 1.364 1.326 1.277 1.245 1.131 1.098 0.999 0.964 0.887 0.873 0.856 0.621
HO
O
N
H
NH
ZH-013
9 8 7 6 5 4 3 2 1 0 ppm
0.80 1.00 1.16 1.47 1.06 1.69 0.87 0.84 1.01 18.94 2.31 3.43 1.00 3.04 22.62 3.75 1.99 4.50 6.56 1.73 2.82

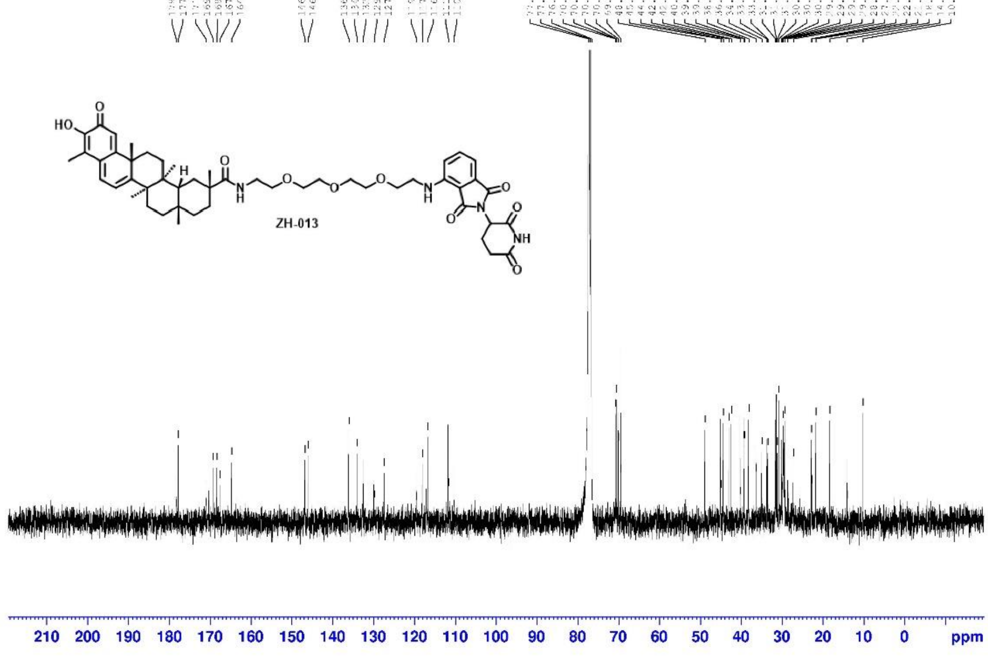
HO
O
N
H
NH
ZH-013
210 200 190 180 170 160 150 140 130 120 110 100 90 80 70 60 50 40 30 20 10 0 ppm

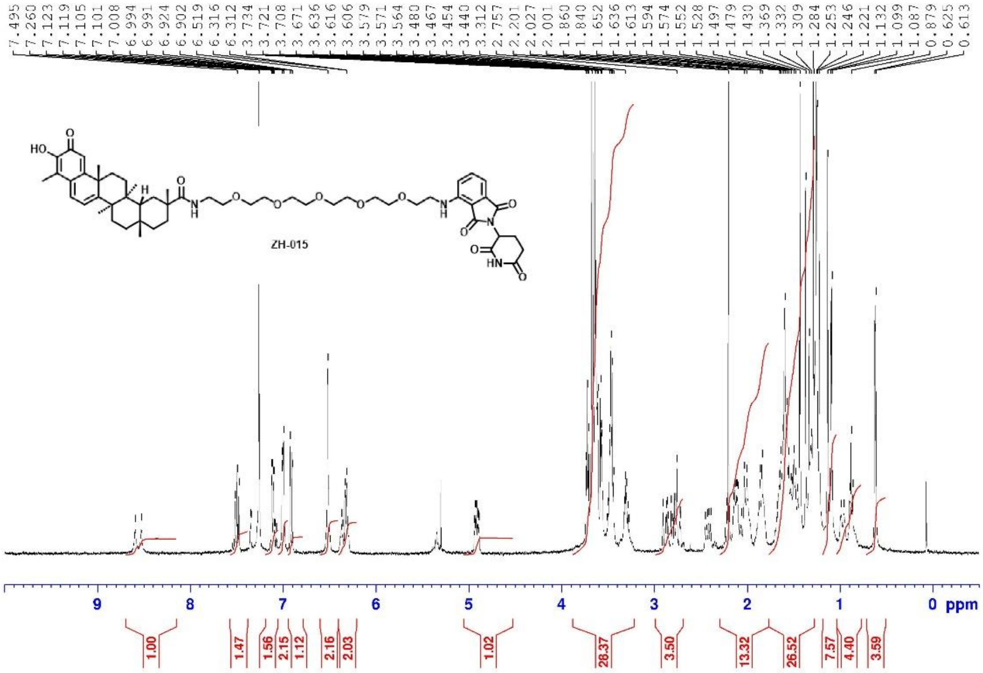
7.495
7.260
7.123
7.119
7.105
7.101
7.008
6.994
6.991
6.924
6.902
6.519
6.316
6.312
3.734
3.721
3.708
3.671
3.636
3.616
3.606
3.579
3.571
3.564
3.480
3.467
3.454
3.440
3.312
2.757
2.201
2.027
2.001
1.860
1.840
1.652
1.636
1.613
1.594
1.574
1.552
1.528
1.497
1.479
1.430
1.369
1.332
1.309
1.284
1.253
1.246
1.221
1.132
1.099
1.087
0.879
0.625
0.613
HO
O
N
H
O
O
O
O
O
N
H
O
N
O
O
HN
O
ZH-015
9
8
7
6
5
4
3
2
1
0 ppm
1.00
1.47
1.56
2.15
1.12
2.16
2.03
1.02
28.37
3.50
13.32
26.52
7.57
4.40
3.59

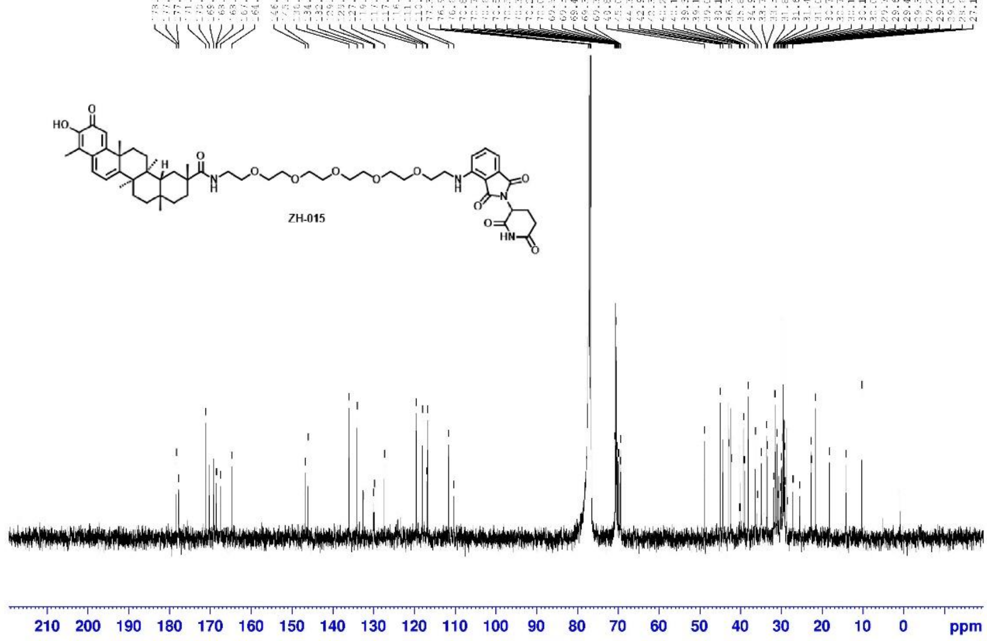
HO
O
N
H
O
O
O
O
O
N
H
O
N
O
O
HN
O
ZH-015
210
200
190
180
170
160
150
140
130
120
110
100
90
80
70
60
50
40
30
20
10
0
ppm

Source data of Fig. 3B and Supplementary Fig. 2

ZH-002

Actin-1 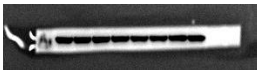 Actin-2 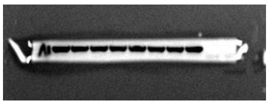

Actin-3 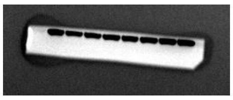

CHK1-1 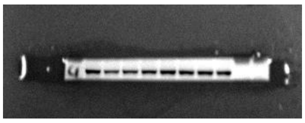 CHK1-2 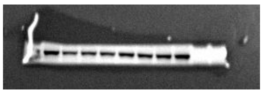

CHK1-3

IKKβ-1 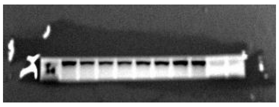 IKKβ-2 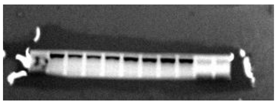

IKKβ-3 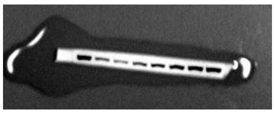

PI3Kα-1 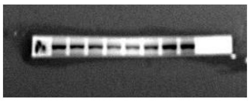 PI3Kα-2 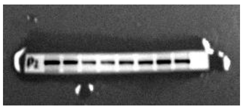

PI3Kα-3 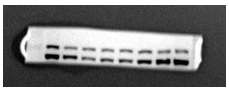

ZH-011:

Actin-1 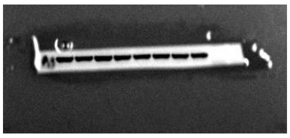 Actin-2 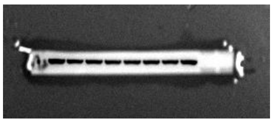

Actin-3 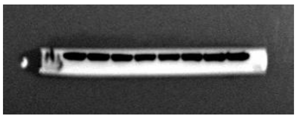

CHK1-1 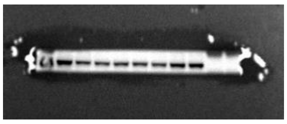 CHK1-2 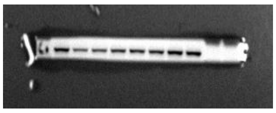

CHK1-3 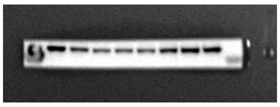

IKKβ-1 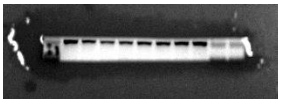 IKKβ-2 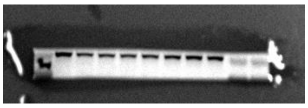

IKKβ-3 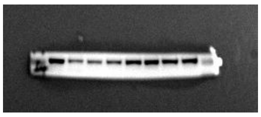

PI3Kα-1 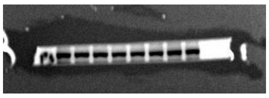 PI3Kα-2 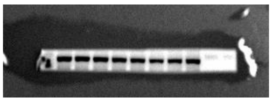

PI3Kα-3 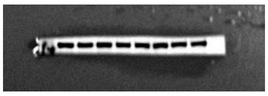

ZH-013:

Actin-1 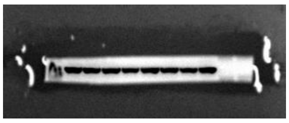 Actin-2 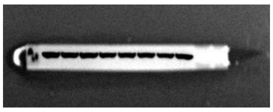

Actin-3 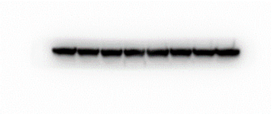

CHK1-1 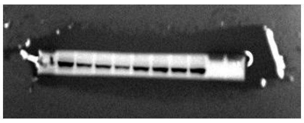 CHK1-2 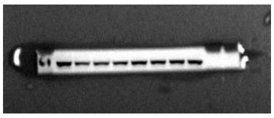

CHK1-3 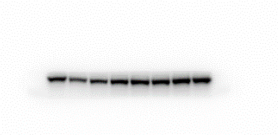

IKKβ-1 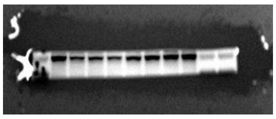 IKKβ-2 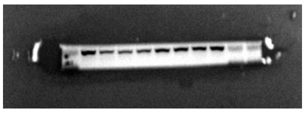

IKKβ-3 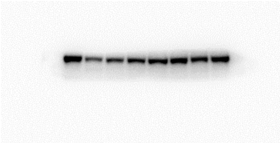

PI3Kα-1 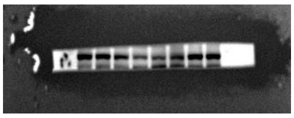 PI3Kα-2 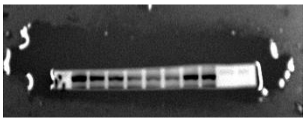

PI3Kα-3 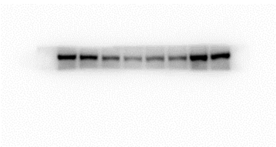

ZH-015:

Actin-1 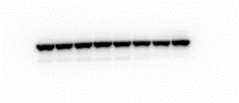 Actin-2

Actin-3 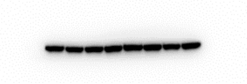

CHK1-1 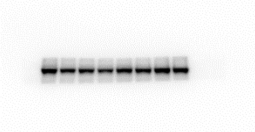 CHK1-2 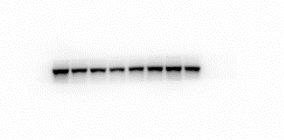

CHK1-3 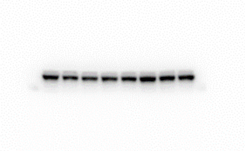

IKKβ-1 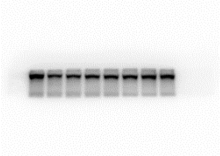 IKKβ-2 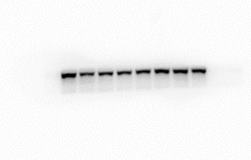

IKKβ-3

PI3Kα-1 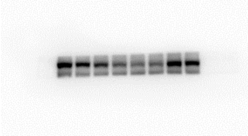 PI3Kα-2 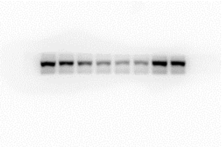

PI3Kα-3 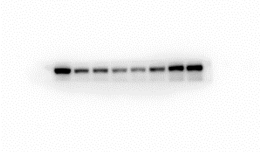

Source data of Fig. 3C and Supplementary Fig. 3

ZH-011:

Actin-1 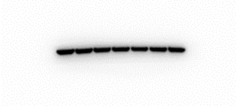 Actin-2 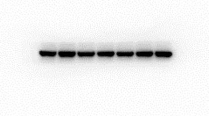

Actin-3 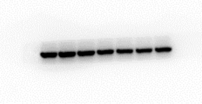

CHK1-1 CHK1-2

CHK1-3

IKKβ-1

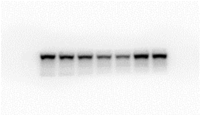

IKKβ-2

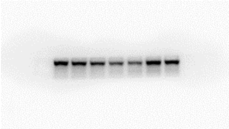

IKKβ-3

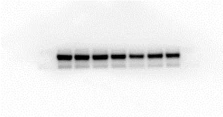

PI3Kα-1

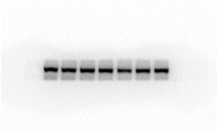

PI3Kα-2

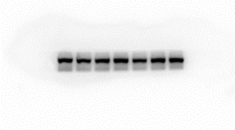

PI3Kα-3

ZH-015:

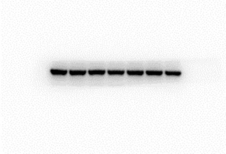

Actin-1

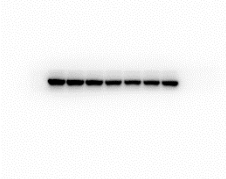

Actin-2

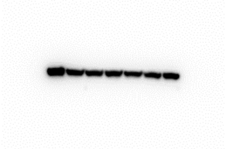

Actin-3

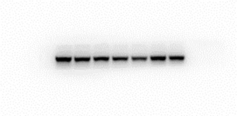

CHK1-1

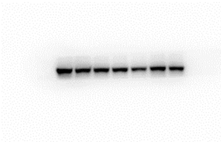

CHK1-2

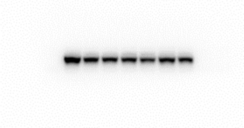

CHK1-3

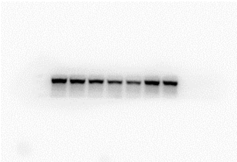

IKKβ-1

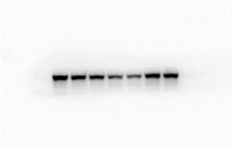

IKKβ-2

IKKβ-3
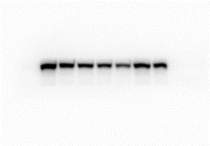

PI3Kα-1
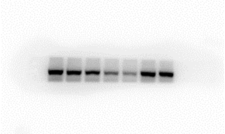
PI3Kα-2
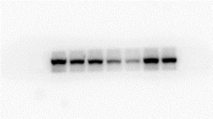

PI3Kα-3
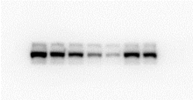

Source data of Fig. 4A

Actin-293T
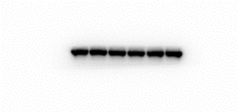
Actin-HepG2
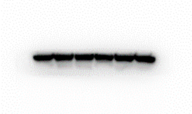

IKKβ-293T
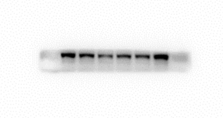
IKKβ-HepG2
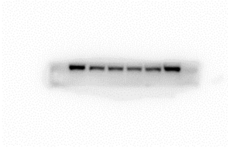

PI3Kα-293T
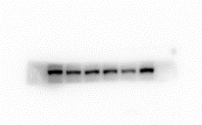
PI3Kα-HepG2
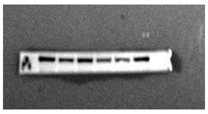

Source data of Fig. 4B

Actin
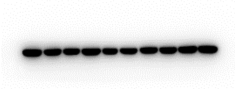

IKKβ
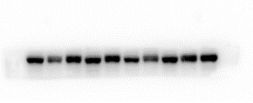

PI3Kα
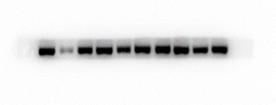

Source data of Fig. 4C

Actin

FLAG-CRBN (input)
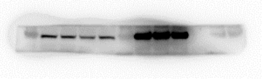
FLAG-CRBN (IP)
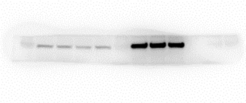

IKKβ (input)

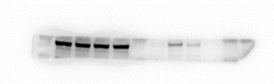

IKKβ (IP)

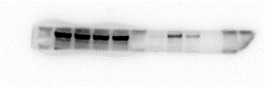

PI3Kα (input)

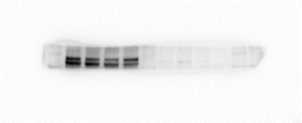

PI3Kα (IP)

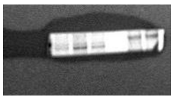

Source data of Fig. 4D, 5C, 6C and Supplementary Fig. 8

PI3Kα-Cela-1

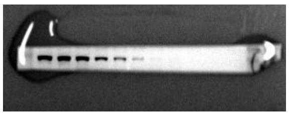

PI3Kα-DMSO-1

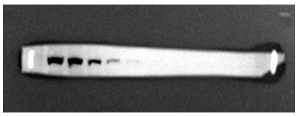

PI3Kα-Cela-2

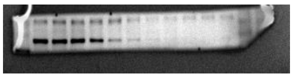

PI3Kα-DMSO-2

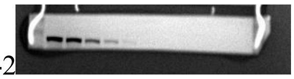

PI3Kα-Cela-3

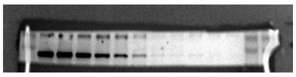

PI3Kα-DMSO-3

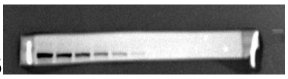

IKKβ-Cela-1

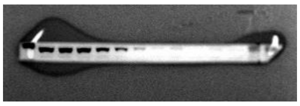

IKKβ-DMSO-1

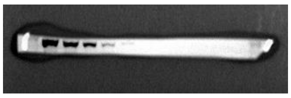

IKKβ-Cela-2

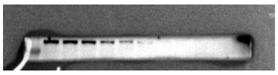

IKKβ-DMSO-2

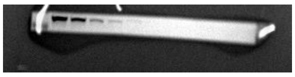

IKKβ-Cela-3

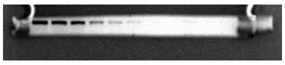

IKKβ-DMSO-3

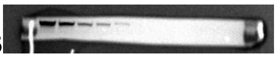

CHK1-Cela-1

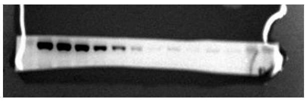

CHK1-DMSO-1

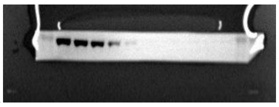

CHK1-Cela-2

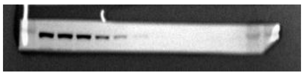

CHK1-DMSO-2

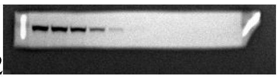

CHK1-Cela-3

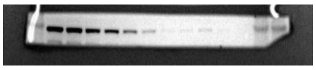

CHK1-DMSO-3

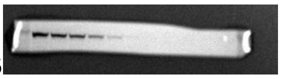

OGA-Cela-1

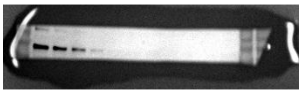

OGA-DMSO-1

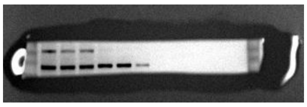

OGA-Cela-2

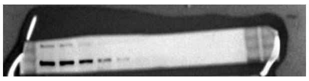

OGA-DMSO-2

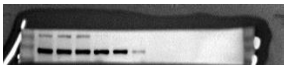

OGA -Cela-3

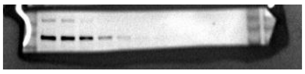

OGA-DMSO-3

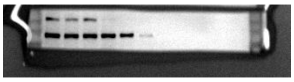

Source data of Fig. 5A

Actin-293T

CHK1-293T

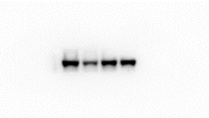

Actin-K562

CHK1-K562

Source data of Fig. 5B

Actin 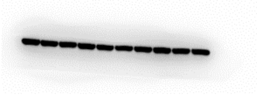

CHK1 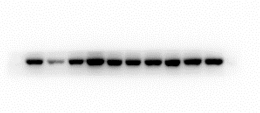

Source data of Fig. 5D

Actin 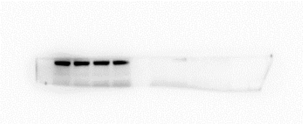

FLAG-CRBN (Input) 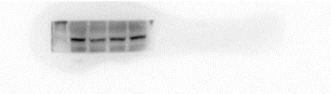

FLAG-CRBN (IP) 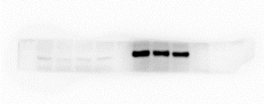

CHK1 (Input) 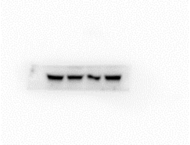

CHK1 (IP) 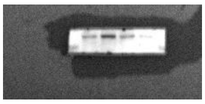

Source data of Fig. 5F

Actin

CHK1 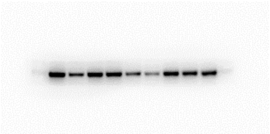

pCHK296 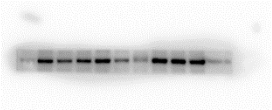

pCHK345 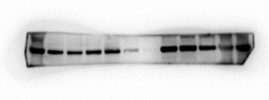

γ-H2AX 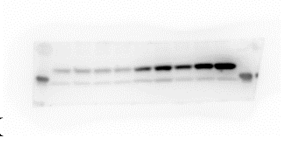

Source data of Fig. 6A

Actin

OGA 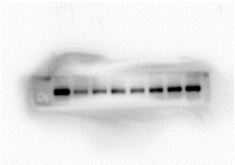

Source data of Fig. 6B

Actin-293 OGA-293 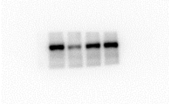

Actin-HepG2 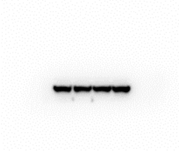 OGA-HepG2 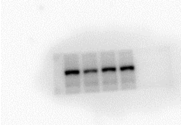

Actin-K562 OGA-K562 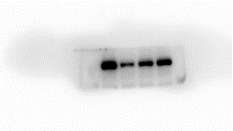

Actin-THP1 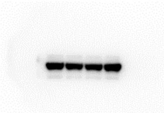 OGA-THP1 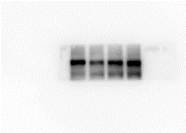

Source data of Fig. 6D

Actin

FLAG-CRBN (Input) 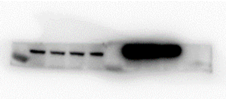 FLAG-CRBN (IP)

OGA (Input) 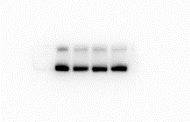 OGA (IP) 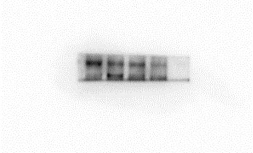

Source data of Fig. 6G

Actin 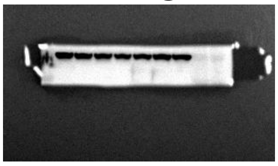

ERCC6L 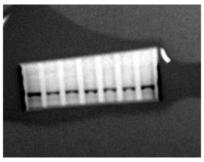 CIP2A 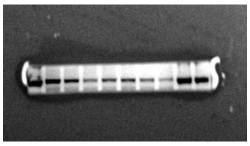

Source data of Fig. 6I

Actin 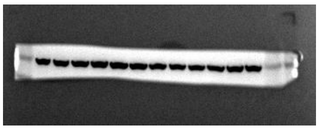

OGA 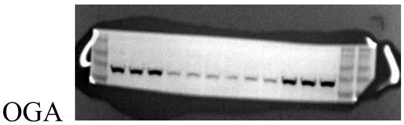 PI3Kα 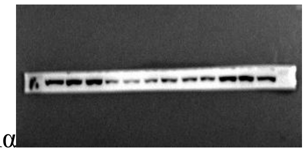

IKKβ 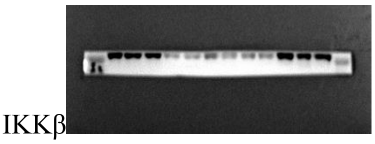 CHK1 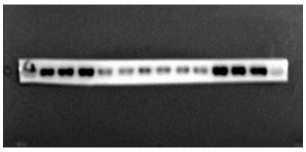

Source data of Supplementary Fig. 4A

Actin 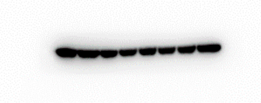 PI3Kα 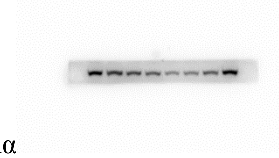

PI3Kβ 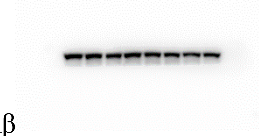 PI3Kγ 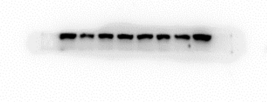

Source data of Supplementary Fig. 4B

ACTIN 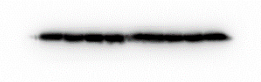 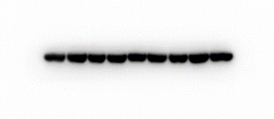

CHK1

IKKβ 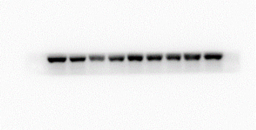 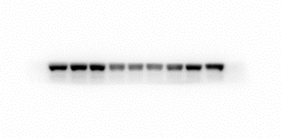

PI3Kα 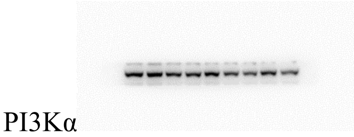

Source data of Supplementary Fig. 5A

Actin 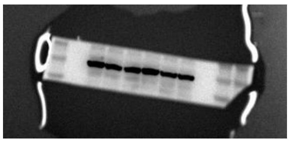 FLAG-DCAF16 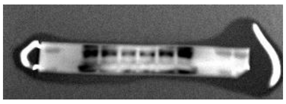

Source data of Supplementary Fig. 5B

Actin

NEK7
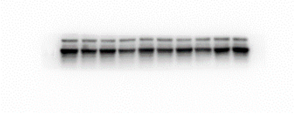

OGA
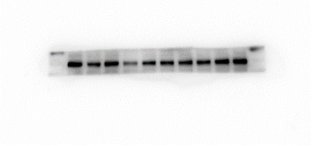

Source data of Supplementary Fig. 5C

Actin
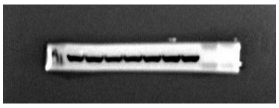

NR4A1
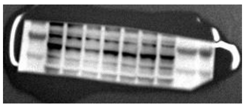

Source data of Supplementary Fig. 6A

Actin
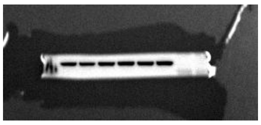

OGA
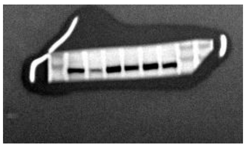

IKKβ
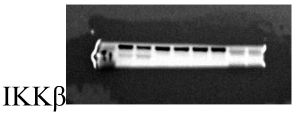

CHK1
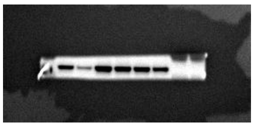

CRBN
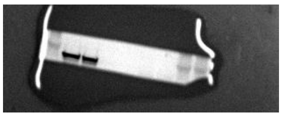

Source data of Supplementary Fig. 6B

Actin
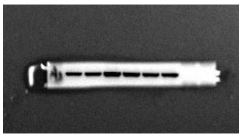

PI3Kα
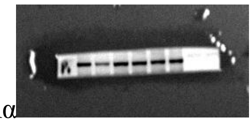

IKKβ
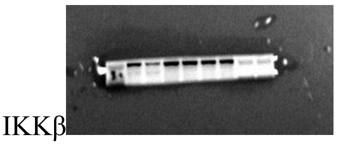

CRBN
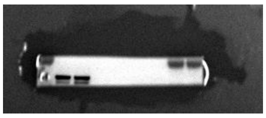

Source data of Supplementary Fig. 7A

Actin

FLAG-CRBN (Input)
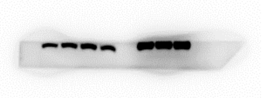

FLAG-CRBN (IP)

IKKβ (input)
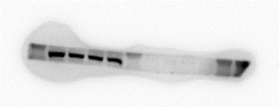
IKKβ (IP)
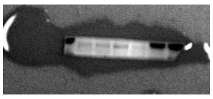
PI3Kα (input)
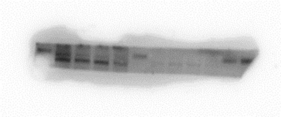
PI3Kα (IP)
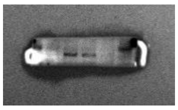

Source data of Supplementary Fig. 7B

Actin
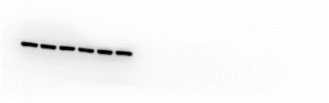
FLAG-CRBN (Input)
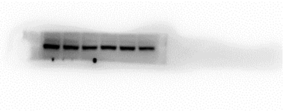
FLAG-CRBN (IP)
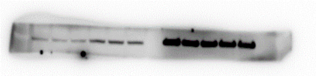
CHK1 (input)

CHK1 (IP)
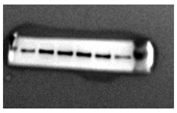
IKKβ (input)
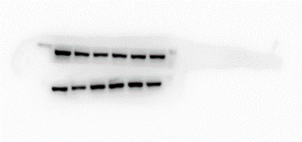
IKKβ (IP)
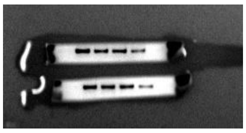
PI3Kα (input)
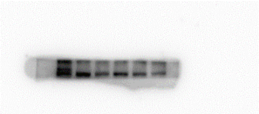
PI3Kα (IP)
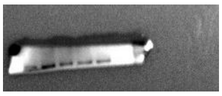

Supplement: Supplementary file 1 — Supporting Information [file ADVS-11-2308186-s003.pdf]
